# Resolving root causes of experiment discrepancies guided by machine learning

**DOI:** 10.1038/s41467-026-76798-5

**Published:** 2026-08-27

**Authors:** D. Neudecker, K. J. Kelly, S. A. Vander Wiel, A. D. Carlson, M. J. Grosskopf, D. A. Brown, B. Pritychenko, N. A. W. Walton, R. C. Haight

**Affiliations:** 1 Los Alamos National Laboratory, Los Alamos, NM USA; 2 National Institute of Standards and Technology, Gaithersburg, MD USA; 3 Brookhaven National Laboratory, Upton, NY USA

**Keywords:** Experimental nuclear physics, Scientific data, Statistics

## Abstract

Scientists rely on accurate experimental data to explain nature and then harness this knowledge for applications addressing human needs. However, discrepancies between experiments of the same observable can impede scientific progress if one does not understand the underlying causes. Here, we developed a process that unravels data discrepancies by first using Bayesian machine learning to relate discrepancies to few of many, potentially biasing metadata features that encode experiment procedures. This machine learning output guides human experts to study discrepancy causes by simulating suspicious aspects of historical experiments or designing modern ones to address open questions. The study findings then lead to rejecting or correcting historical data on firm scientific bases. This process is demonstrated for the energy spectrum of neutrons emitted promptly (<1 ns) after fission of ^252^Cf, a trusted nuclear physics Standard. It reduces the spread in experimental ^252^Cf spectra by up to a factor of 6.

## Abstract (web summary)

The authors present a framework to identify and resolve discrepancies between experimental datasets. Combining Bayesian machine learning with expert analysis, they uncover hidden sources of bias in historical measurements.

## Introduction

Progress in most scientific disciplines relies on accurate measurements. Resulting experimental data or observations are key to understanding fundamental principles of nature, calibrating models of these natural processes, and then harnessing our resulting understanding to craft new applications that address human needs. Examples of subject areas that rely on accurate experimental data or observations to address society’s most pressing challenges are accurate forecasting of extreme weather phenomena^1^ and infectious disease spread^2^ and tackling society’s mounting energy needs by predictive simulations of new designs for fusion and fission reactors^3^.

**Fig. 1:**
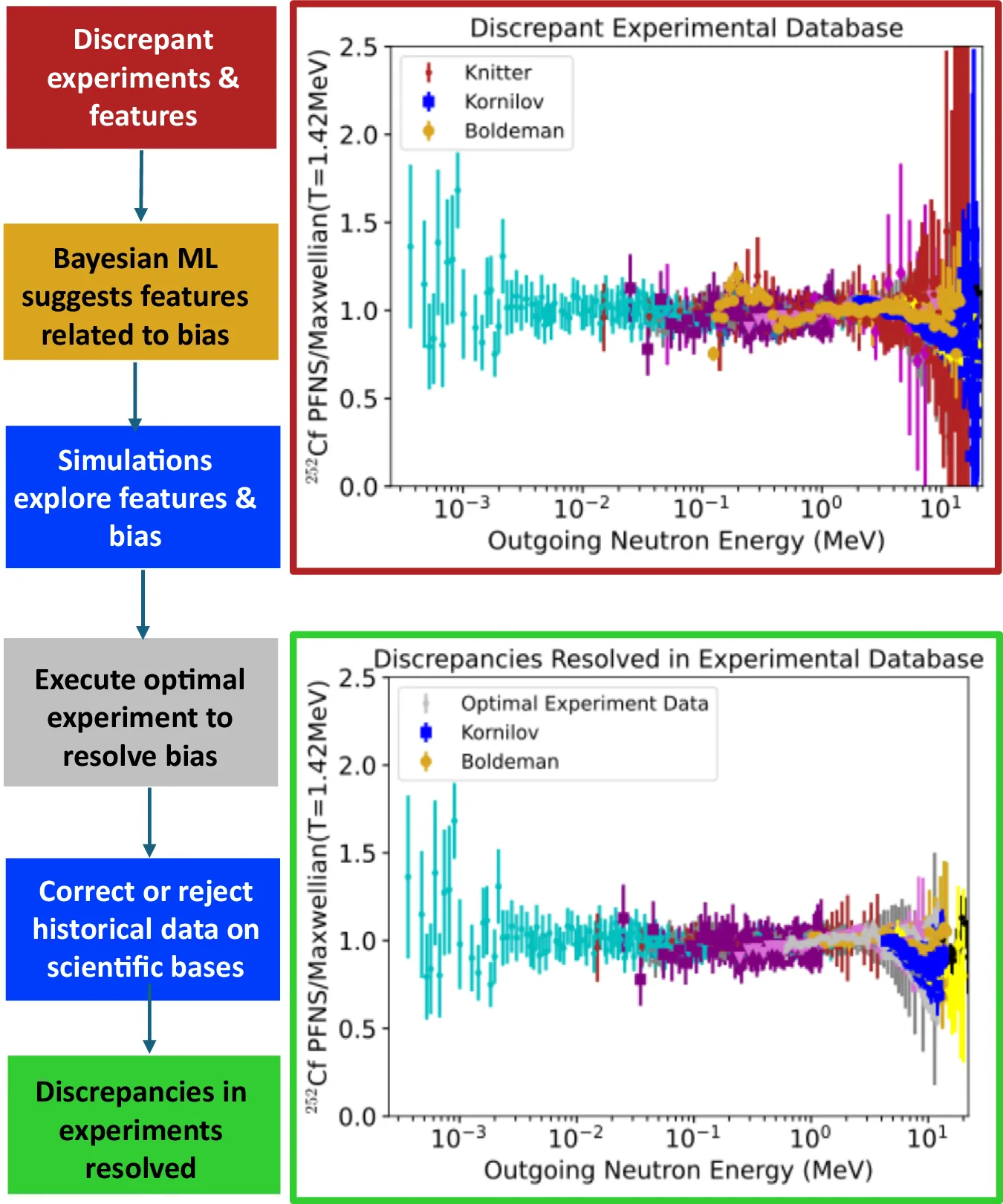
Schematic representation of process to resolve discrepancies in an experimental database. Starting from discrepant experimental data (top), ML (machine learning) methods provide clues as to what their root cause could be. These clues then trigger simulations correcting historical data and designs of new experiments providing further validation. Thus, we attain our goal to resolve discrepancies in the database (bottom) that ultimately fuels scientific progress. Only those datasets are called out by name in the legend that are relevant for this work. The abbreviation PFNS means prompt fission neutron spectrum and the variable *T* is the temperature of a Maxwellian function, $\sqrt{E}*\exp (-E/T)*(2/\sqrt{\pi }/\sqrt{T}/T)$, with outgoing neutron energy *E*.

These applications rely on accurate numerical model predictions to approximate nature. However, these models are only as reliable as the experimental data used for their calibration. One critical barrier to progress in science arises when multiple measurements of the same observable differ systematically and distinctly beyond their uncertainties despite the experiments being performed with commonly used techniques by renowned teams that trust their respective results. Even more crippling to scientific progress is that researchers may lack an understanding of the root causes of these discrepancies^4^. As an example, the top of Fig. 1 shows color-coded experimental data sets that differ by substantially more than their 1-sigma uncertainty bands. In fact, it is unclear from this collection of experimental data (referred to as a database) what the underlying true curve should be, especially at high x values.

Without knowing the root causes of discrepancies, scientists do not know which experimental data to trust. Such experimental-data inconsistencies result in uncertainties on model parameterizations or estimates of the true curve for a natural phenomenon that are based on these experiments as input. Ultimately, these unquantified uncertainties limit applications of science. For example, we cannot accurately forecast the location where an extreme weather phenomena might occur, how quickly a disease will spread, or whether a new nuclear reactor design will be feasible, economical, and safe.

In this work, we show, as a proof-of-concept within the domain of nuclear physics, how the AIACHNE (AI/ML Informed cAlifornium CHi Nuclear data Experiment) team resolved detrimental experimental discrepancies. To this end, we applied machine learning (ML) methods to answer the question: “What are the root causes of discrepancies between seemingly trustworthy experiments that measure a common observable?” At the beginning of the process we are faced with a database of experiments in the top of Fig. 1 that has a large unexplained spread among several data sets. We then utilize a Bayesian model to highlight metadata features that are unique to discrepant data. These metadata hold clues to the scientific root causes of discrepancies that can be unraveled by targeted follow-up simulations of historical experiments and/ or new measurements aimed specifically at probing suspected sources of disagreement. At the conclusion of this process, the resulting modified database (bottom of Fig. 1) has a distinctly narrower spread by up to a substantial factor of 6 because we could reject or correct (and thus salvage) historical data on sound scientific bases or substantiate them with a new experiment. Our specific method applies to finding root causes of discrepancy in data sets wherein one quantity is measured as a function of another quantity and it requires collection of metadata features associated with each experiment. For instance, an observable could be measured as a function of time or temperature with metadata describing the measurement instrument and location. Given these general input requirements, the approach presented here could be tested for use in subject areas with similar data structures.

## Results

### Process to unravel root cause of discrepancies

We answer the question “What are the root causes of discrepancies between seemingly trustworthy experiments?” by the process shown in Fig. 1. The first step is to collect discrepant experimental data and metadata features that are fed in the second step into a Bayesian ML model described in Section “The Bayesian Model”. The hypothesis here is that biases in measured data must stem from something inherent to the experiment—for instance, poorly understood equipment or incorrect analysis steps. Therefore, metadata encodes intricate details of experiment makeup and analysis.

The second step of the process uses a Bayesian model to determine for each feature category a posterior distribution for the strength of a potential bias. This posterior distribution has different values for each feature value in a category. These results thus inform us which metadata values in a specific category are more likely to be related to bias.

We chose a horseshoe prior to sample the strength of potential biases with the Bayesian model and link them to specific metadata feature values as it enforces sparseness so that not every statistical fluctuation is identified as bias and a single root cause is preferred to multiple interacting ones^5^. The fitted Bayesian model highlights a sparse set of features that are potentially responsible for biases^6^. Because we obtain posterior distributions for each feature category at a time and several of the feature categories might be correlated with another, the same bias might be attributed to be related to values of a few feature categories. Therefore, it is vital that subject matter experts further interpret these posterior distributions.

Therefore, in the third step of the process, experts investigate these posterior distributions as clues (rather than fact). One way to investigate the ML findings is by simulating the aspects of historical experiments to explore whether purported reasons for data discrepancies suggested by the Bayesian model can be grounded in physical truth. The conclusions drawn from these simulations then guide what would be the most informative new experiment to answer any remaining open questions on key drivers of data discrepancy. In doing so, scientists can optimally select the most impactful experiment to reliably reduce potential biases in data, thus effectively using available funds to reduce the most pertinent data uncertainties.

In the second-to-last step, all information gained from simulations and the new experiment is gathered to (a) reject, (b) correct, or (c) validate historical data based on sound scientific reasoning. Finally, this produces a database in which discrepancies are resolved (bottom of Fig. 1).

Execution of this process requires the following basic data inputs:

- Discrepant measurement values of one quantity of interest as a function of another along with their uncertainties and correlations. Uncertainties should encompass all known sources of imprecision and potential bias but not be artificially enlarged to cover discrepancies.
- Metadata features that uniquely encode how the data were physically measured (e.g., detector type or other aspects of the experiment environment) and analyzed (e.g., corrections for known experiment biases). Metadata can be continuous (e.g., environment temperature) or discrete (like detector type). It is key that one defines at first all feature categories that could lead to biases in the studied data to enable the Bayesian model identifying biases. In addition, one must guarantee that metadata are easily interpretable by the Bayesian model, which requires utilizing a nomenclature to catalog feature categories and names that is consistent across all data sets. If for a specific experiment, the value of a feature category is either unknown or not applicable, this should be stated as such. Data sets where large parts of the feature values are unknown, especially if the most important values are all unknown, should be rejected. Creating such a consistent database can be a time-consuming aspect of the meta-analysis.

### Application to nuclear spectra

We demonstrate this process to understand biases in experimental data that inform the estimate of the ^252^Cf prompt fission neutron spectrum (PFNS) from a collection of 21 experiments^6,7^ performed over the 45-year period from 1973 to 2017. Fission describes a process in which a heavy nucleus (in this case ^252^Cf) splits into few (often only two) fragments of lower mass and concurrently releases neutrons along with a copious amount of energy. The PFNS is the energy distribution of promptly emitted, that is within <1 ns, neutrons from fission; thus a PFNS curve is a one-dimensional function of outgoing neutron energy. Quantitative knowledge of the ^252^Cf PFNS is, for instance, exploited to monitor the fluence of neutrons in a nuclear fission reactor^8^, detect hidden radioactive material in cargo^9^, and treat specific types of cancer^10^. The PFNS for ^252^Cf is also particularly important because it is defined as a Standard^11^ by the International Atomic Energy Agency, which means that this entity is highly trusted and can be reliably used to inform many other fission measurements through detector calibration. So, accurate knowledge of the ^252^Cf PFNS with small and defensible uncertainties positively impacts a wide array of applications.

### Database studied here

^252^Cf PFNS is probed in composite experiments, meaning that several analysis steps and auxiliary measurements are required to transform the directly measured quantities (neutron and fission counts) to the desired underlying physics quantities (the ^252^Cf PFNS function over a range of outgoing neutron energies)^12–14^. Example pictures of hardware used to perform these experiments can be found for instance in refs. ^15–17^: There, a central chamber detects the occurrence of fission events while an outer detector array detects their promptly emitted neutrons. The time between a given fission and an associated neutron detection is converted to energy of the emitted neutron (the horizontal axes of plots in Fig. 1) as described in Section “PFNS Experiment Analysis“. Vertical axes relate to probabilities that neutrons are emitted at given energies and are the result of complex analyses briefly summarized in Section “PFNS Experiment Analysis”.

Metadata were collected to encode details of the measurement equipment and analysis techniques of these composite experiments for 41 feature categories across 21 distinct data sets^6^. Each feature category consists of several different levels (e.g., different detector types) that are cast into a common nomenclature that is consistent across all experiments. Typical features that describe the analysis techniques include whether backgrounds were removed or how the detector response was assessed, while equipment features record details such as the type of detectors utilized. Experimental mean values were extracted from the openly available experimental database EXFOR^18^. The uncertainties for all known effects were first estimated based on EXFOR data and the literature tied to each experiment. Finally, uncertainties missed by the authors were augmented based on physics knowledge on PFNS experiments^13^. For instance, no uncertainties for impurities in the sample, that could result in a mixture of PNFS from several materials rather than just ^252^Cf, were found for the experiment related to ref. ^19^. Hence, covariances for this (small) effect were estimated based on a broad review of PFNS experiment uncertainty^13^ and added in quadrature to the known covariances for this measurement.

**Fig. 2:**
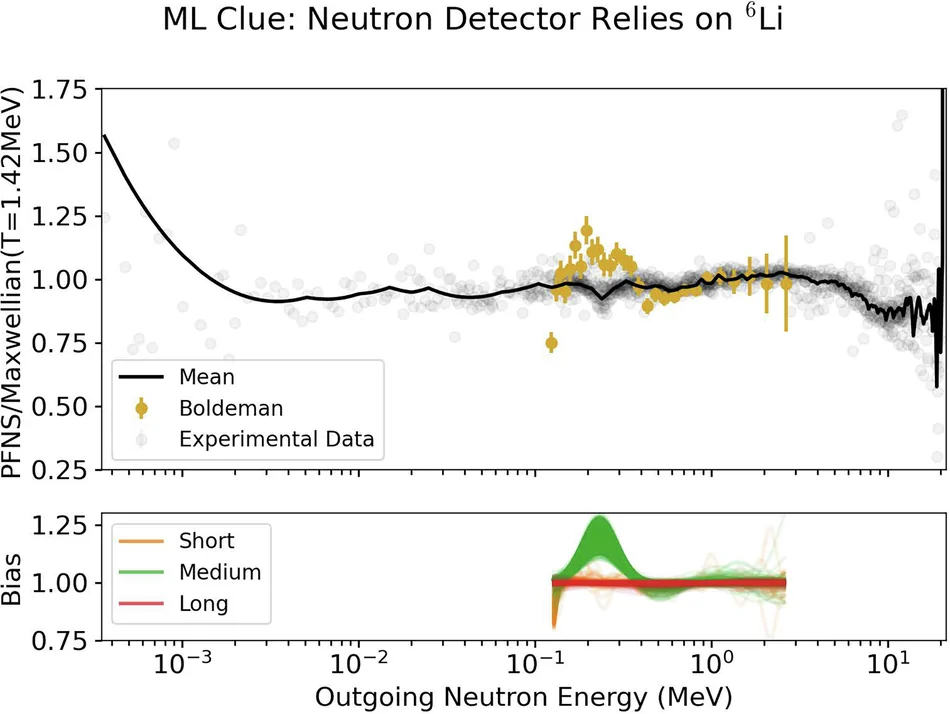
The Bayesian model correctly identifies the physics source of bias in Boldeman data. It is shown that the Bayesian model correctly relates the bias in Boldeman data (dark golden) to the neutron detector containing ^6^Li. All other experimental data are given in light gray. The bias functions are shown in the bottom boxes of the figures along with their potential sources of bias in the title of the plot. This bias could be of short (orange), medium (green) and long (red) energy range as described in Section “The Bayesian Model”. The mean of the bulk of the data as calculated according to Eq. (6) is shown as a black solid line.

It is evident that there are significant discrepancies between the different data sets in the top of Fig. 1. For instance, the data of Boldeman et al.^20^ show in Fig. 2 a significant increase close to 0.2 MeV compared with the bulk of the data. In fact, the bottom of Fig. 2 shows that the Bayesian model identified a significant medium-range bias term for Boldeman data compared to the mean estimated from the entire database. The reason for this bias is understood by subject matter experts^7^ as will be described below. However, it was unclear at the beginning of the project how to best infer the ^252^Cf PFNS from 8–20 MeV. Some experimental data vary by up to an unacceptable 50% in each direction from the bulk of the data. The largest variations can be observed in the data by Knitter^21^ and Kornilov^22^ that are studied below. The key issue is not only the large spread in the data but also that we lack the knowledge of the main physics effect driving these discrepancies; above 8 MeV, these discrepancies could be related to the neutron or the fission detector response, incorrect background removal, incomplete discrimination of neutrons and *γ* rays, or a combination of these. In short, we are truly faced with large discrepancies in data with unknown cause from 8 to 20 MeV, which we will resolve below.

### Clues found by ML and decisions taken

#### Example validating the Bayesian model

We used the experimental data and metadata of Boldeman et al.^20^ to test if the Bayesian model correctly identifies the source of a known discrepancy. The discrepancy of Boldeman data from 0.12–0.4 MeV is related to the nuclear reaction used to detect neutrons in this energy range: the ^6^Li(*n*,*t*) reaction in which a neutron is captured by a ^6^Li nucleus and releases a tritium nucleus. At 0.2 MeV, the ^6^Li(*n*,*t*) reaction has a peak in the cross section that will distinctly bias the ^252^Cf PFNS if the detector response is not perfectly understood. It is shown in Fig. 2 that indeed the Bayesian model locates a bias in Boldeman data close to 0.2 MeV (lower-left panel) that is related to using a ^6^Li-based neutron detector as called out in the title^6^. Several other potential feature values were called out as explanation for the bias, the majority of them was tied to the neutron detector (such as how its response was determined, or scattering effects distorting this response). While the AIACHNE team attempted to correct these data for this feature, unfortunately Boldeman could not provide the detailed information necessary to amend the detector response. Hence, the portion of Boldeman data measured with the ^6^Li-based neutron detector was rejected as biased for the final accepted database in the bottom of Fig. 1. This example demonstrates that the Bayesian algorithm works as expected: it identifies the correct source of bias suspected by the human expert. The added benefit of the Bayesian model is that the algorithm defines the location and strength of the bias that could be used to model the bias for subsequent data analysis.

#### Example where Bayesian model clues lead to correcting data

**Fig. 3:**
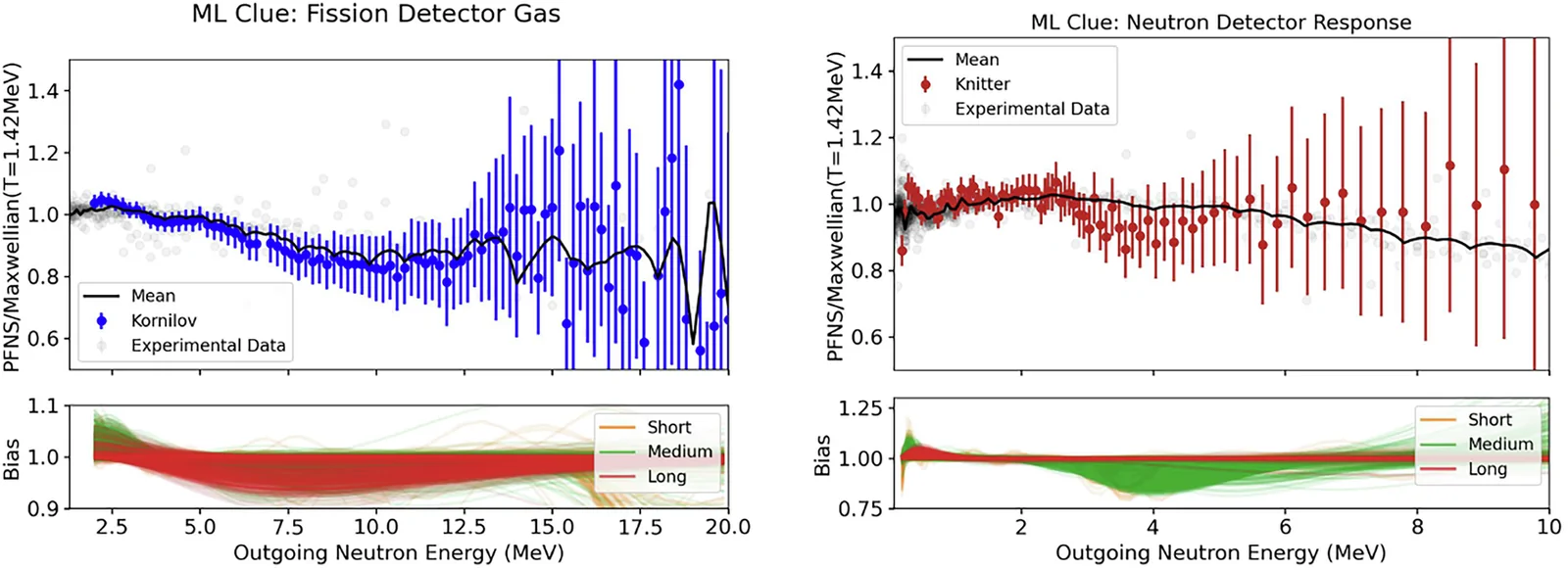
Two figures highlight sources of bias for Kornilov (left) and Knitter (right) data. Biases found by the Bayesian model for Kornilov (blue)^22^ (left) and Knitter data (dark red)^21^ (right). The bias functions are shown in the bottom boxes of the figures along with their potential sources of bias in the title of the plot. This bias could be of short, medium and long energy range as described in Section “The Bayesian Model”. The upper boxes show the data in comparison to the bulk of other experiments in light gray to not distract from the data under investigation. The mean of the bulk of the data as calculated according to Eq. (6) is shown as a black solid line as an eye-guide for discrepancies of the plotted data to the bulk of the data.

Kornilov data^22^ have a significant spread and deviation from other data above 10 MeV. The Bayesian model highlighted three possibilities for issues: (1) in the fission fragment detection efficiency (see left-hand side of Fig. 3 where ML points to the gas used in the fission detector), (2) the background correction, or (3) the neutron detector response. Relying on the ML findings alone would have led to inconclusive results in as far as one does not know which ones of these three (very different) potential issues could be the sources of bias. Here, it was key that human experts followed up with a step-by-step analysis of all raised issues with a frequent collaborator of Kornilov^7^. In the end, it was found that the key issue in the data at highest and lowest energies is related to biased data (for the light output) assumed for the analysis of the neutron detector response and an imperfect choice of the neutron detector bias setting for part of the data. Data were corrected for the latter effect and truncated to an energy range where the former issue is negligible^7^ to yield the revised data set shown in the top of Fig. 1. Thus, we were able to salvage the data, and implicitly the funds used for the measurement, for a now unbiased use in informing the ^252^Cf PFNS. It should be emphasized that the key that led to salvaging the data was the limited number of possibilities identified by the Bayesian model. Without those clues, one would still be confronted with a misleadingly long list of potential biasing factors in a general ^252^Cf PFNS measurement.

#### Example where Bayesian model clues lead to rejecting data

The data set of Knitter^21^ showed large biases above 3 MeV in the right-hand side of Fig. 3. These were attributed by the Bayesian model to issues in the neutron detector response, background correction, and neutrons losing energy from interactions in the environment before hitting the neutron detector. Previous simulations of the latter effect for related Knitter experiments^23^ have highlighted this effect as a source of bias. Additionally, a re-investigation of Knitter literature (prompted by the ML clues) revealed that there were indeed simplifying assumptions made for the detector response and background subtraction for this data set. These many major issues could not be conclusively corrected because of lack of measurement information. However, there is now a clear understanding of what effects bias this measurement, and thus a sound scientific reason for rejecting the data. The Bayesian model provided the crucial starting point for experts to then verify the source of measurement biases via simulations of salient parts of the measurements. Without the clues of the Bayesian model, we would still grapple with the question of whether it is justified to reject these data.

### Optimal experiment

All three examples shown above have one common denominator when it comes to potential sources of bias, namely: determining the neutron detector response that is described in more detail in Section “PFNS Experiment Analysis”. In fact, this is among the most difficult aspects of a ^252^Cf PFNS analysis when going from raw measured counts to reported PFNS (see Section “PFNS Experiment Analysis”)—in part because the ^252^Cf PFNS itself is frequently used to define the detector response in measurements of other quantities. The measurements in Fig. 1 rely on ^1^H, ^6^Li, or ^235^U nuclear reactions to define the neutron detector response^7^ following Eq. (9). It was shown with the Boldeman example that using the ^6^Li(*n*,*t*) reaction to determine the detector response can bias PFNS results if the response is not carefully analyzed. Hence, the question arose as to how one can trust the other experiments relying on ^1^H and ^235^U reactions. While measurements relying on ^1^H and ^235^U reactions agree, this does not necessarily mean that these measurements are correct. They could just be biased in the same direction resulting in false confidence in the data. Therefore, the goal of our optimal ^252^Cf PFNS experiment was to validate whether experiments relying on ^1^H and ^235^U reactions to determine the detector response can be trusted as this was the major outstanding question that could have led to large biases in data. Our experiment was optimal in as far as it was designed^15^ to be as uncorrelated with past experiments as possible for this particular feature. Hence, neither ^1^H, ^6^Li nor ^235^U reaction were used to determine the detector response. Instead, elastic scattering reactions on ^9^Be and ^12^C were utilized for the very first time for ^252^Cf PFNS to define the detector response in an energy range where the bulk of experimental ^252^Cf PFNS exists. If agreement between data from this new, uncorrelated experiment and historical measurements is found, this would increase our trust in data relying on ^1^H and ^235^U reactions for the detector response analysis. This new measurement, shown in the bottom of Fig. 1, did indeed validate previous data. As this is an independent technique to elucidate the key issue in these types of measurements, it strongly enhances our trust in the remaining historical database where discrepancies were resolved.

### Combining the knowledge gained and assessing impact

**Fig. 4:**
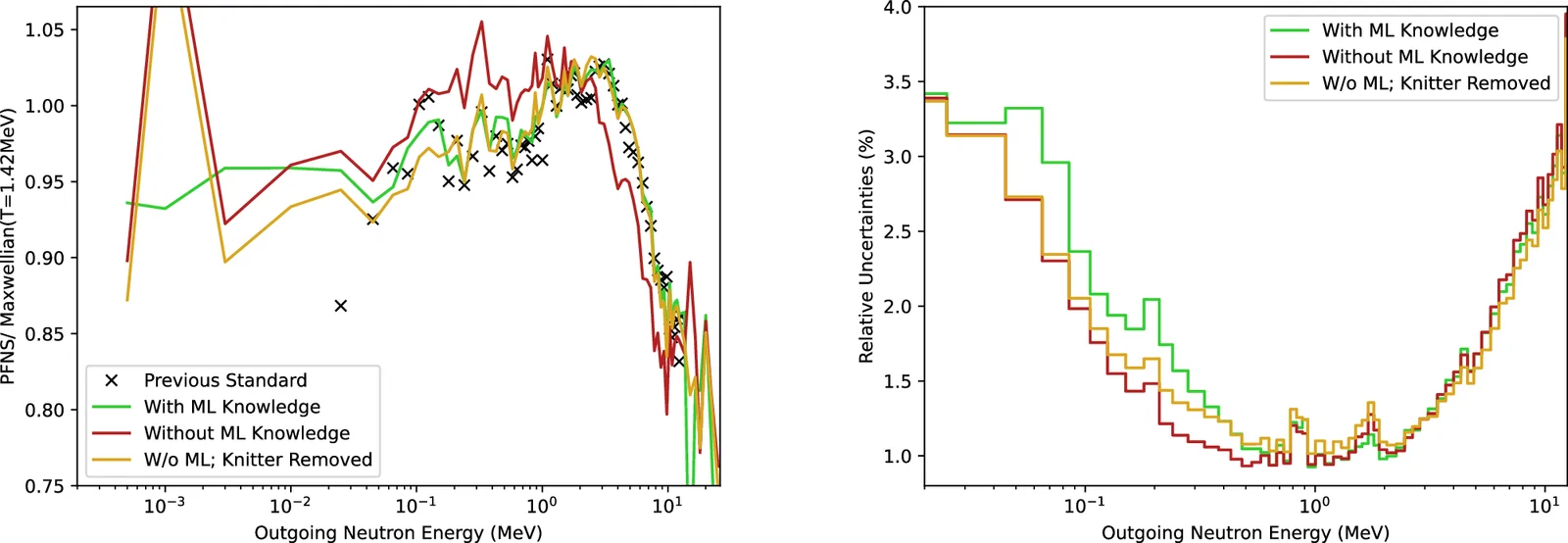
Impact of the findings of the Bayesian model on evaluated means (left) and relative uncertainties (right). Evaluated means (left) and relative uncertainties (right) are shown coming out of regression of the experimental database with (green) and without (dark red) ML knowledge. Means are also compared to the previous Standard of this observable (black “x”). Clearly, using the database without ML knowledge biases the evaluated means strongly and leads to underestimated uncertainties. It is shown that a large part of the bias in evaluated data vanishes if Knitter data are removed from the database (green). These data have been indicated by ML to be biased.

The knowledge gained through this ML-supported process has clear qualitative and quantitative impacts. Qualitatively, we distinctly reduced the spread of experimental data in Fig. 1 starting from the clues obtained by the Bayesian model. If one bins data into histograms following the grid structure in Fig. 4 and assesses the spread between maximum and minium experimental PFNS values for the database after and before the ML process, the spread in data means reduces by an unweighted average of 2.8 across all data and a maximum value of up to 6 (ignoring the two most glaring outliers).

To quantitatively assess the impact of the information gained starting from the Bayesian model, we perform a regression algorithm on the database (mean values and covariances) shown in the top versus bottom of Fig. 1. For this regression, we use generalized least squares factoring in an uninformative prior as well as experimental data and covariances. The resulting data from the regression on all experimental data are referred to as evaluated data in the field of nuclear physics.

Figure 4 shows that the mean calculated from using the database without ML knowledge versus the one with ML knowledge differs drastically. The evaluation with the database before ML is clearly inaccurate as we have an estimate for this observable by the Neutron Data Standards Committee^11,24^. This stark difference is mainly driven by the biased Knitter data; the difference between evaluated means with and without ML knowledge is less pronounced in the left-hand side of Fig. 4 once we remove the data set. Moreover, Knitter data also drive down the evaluated uncertainties below 1 MeV in the right-hand side of Fig. 4, making the evaluation also appear more precise. Hence, Knitter data render the evaluated data more precise but plainly incorrect, which can be dangerously misleading for applications. The Bayesian model provided clues to reject these data with a sound physics justification.

**Fig. 5:**
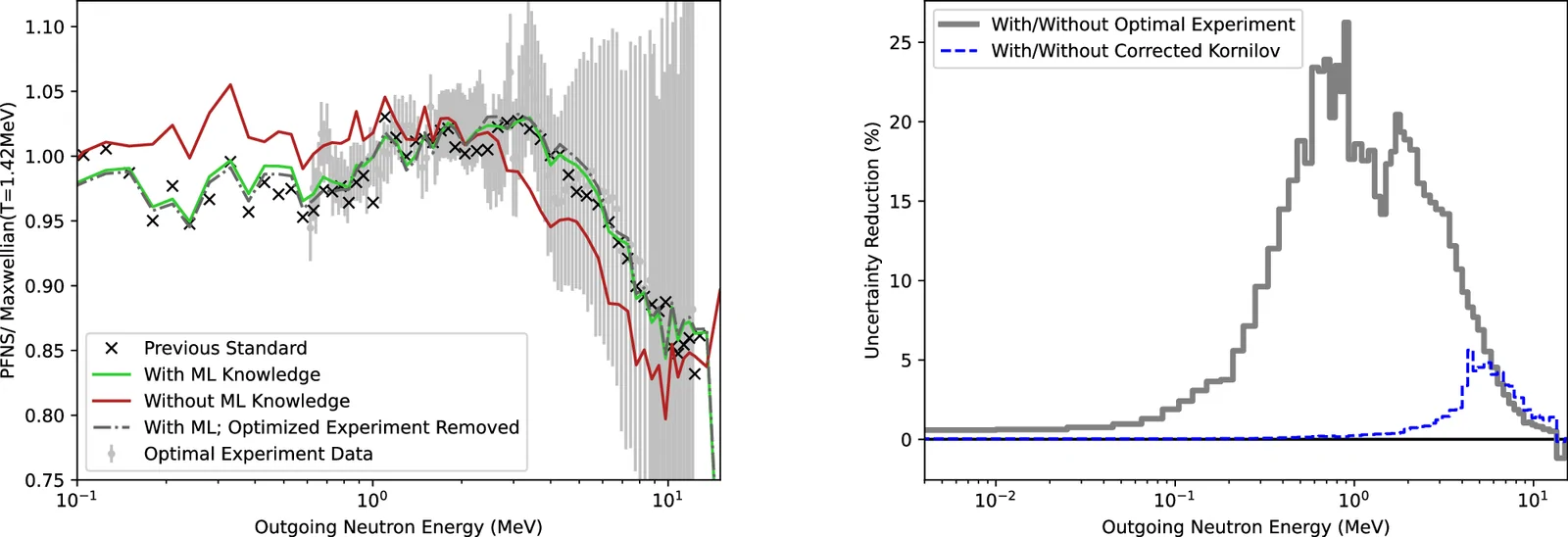
Impact of new experiment and salvaging a past one on evaluated means (left) and uncertainties (right). The impact of the new experiment optimally selected to study a key remaining analysis step (detector response) of this measurement type is shown for mean values (left) and relative uncertainties (right). The new data (light gray, right-hand side) confirm previous experiments and, hence, their detector response treatment; more explicitly, the gray pointed-dashed line, that is obtained including ML knowledge but excluding the optimal experiment, is nearly on top of the green line, which includes ML knowledge and the optimal experiment. The red line shows the very different evaluation when one entirely excludes ML knowledge. Inclusion of the optimal experimental data reduces the evaluated uncertainties by up to 25% (gray solid line in the right-hand side). In the right-hand side it is also shown that salvaging Kornilov data (blue dashed line) reduced the evaluated uncertainties by 5%.

Leaving uncorrected Kornilov data in the database without ML impact (left of Fig. 4, curve “W/o ML; Knitter Removed”) has a less significant impact on the evaluated ^252^Cf PFNS. However, there is a 5% decrease of evaluated uncertainties in the right-hand side of Fig. 5 if we are able to include the corrected data versus rejecting them entirely. Hence, salvaging the data of Kornilov by correcting for newly-discovered effects has a tangible impact on evaluated uncertainties that will propagate to applications ranging from nuclear medicine to reactor physics and safeguards.

Finally, it is shown in the left-hand side of Fig. 5 that the evaluated mean values change very little when including the optimized experimental data. This effect was expected as the new experiment confirmed the trend of previous data. The value of this measurement lies in that it underscores the trust in the historical data in the bottom of Fig. 1 by an independent measurement. This trust can be measured by the evaluated uncertainties decreasing by an additional 25% compared to the database informed by ML but without the new experiment in the right-hand side of Fig. 5. This uncertainty reduction of 25% concerns an energy range that covers 80% of the integral of the PFNS. In summary, this experiment was guided in its objective by the Bayesian model and was successful in answering a key remaining question on the ^252^Cf PFNS and reducing uncertainties.

## Discussion

This work shows how one can pull the thread to unravel the root causes of discrepancies between several historical experimental data sets using machine learning methods. We demonstrate this process as a proof of concept using 21 ^252^Cf prompt fission neutron spectrum data sets; in this case, the machine learning algorithm provided the clues to reduce the spread in the database by up to a factor of six. To this end, a Bayesian model was used to provide clues as to what metadata features are linked to data sets being discrepant from the bulk of the data. These clues are then the starting point for further investigations into what could potentially bias historical data. The investigations’ outcome is to either reject or correct historical data with a firm scientific basis that was jump-started by the Bayesian model’s clues. It should be emphasized that the ability to correct the historical data salvages not only millions of dollars of funding, but can also lead to a reduced overall uncertainty on the quantity of interest when one performs regression on the database. Finally, the Bayesian model clues and resulting targeted investigations of historical experiments are also beneficial to pinpoint what are the key remaining issues in historical data. These issues can then be addressed with a new measurement to further increase trust in a database—reducing uncertainties by 25% in the presented case. In a nutshell, the knowledge gained on ^252^Cf spectra through the process described here leads to more targeted experiments, faster progress in science related to ^252^Cf spectra, and more efficient use of funding.

One could apply this process to subject areas that can generate the required input, namely, several experimental data sets and their uncertainties as a function of a continuous observable as well as descriptive metadata features describing the experiments. The next step in this research is coupling the Bayesian model with a Large Language Model to easily extract and condense metadata features to be AI/ML ready.

## Methods

### The Bayesian model

In an ideal, no-bias world, a Bayesian model for experimental data, χ^*E*^, would be written as:

1 $${{{{\mathrm{\chi }}}}}^{E}={{S}}{{{{\mathrm{\chi }}}}}^{T}+{{{\mathbf{\varepsilon }}}}.$$

There, χ^*T*^ equates to the true PFNS value, while χ^*E*^ is the observable distorted by random error, **ε**. The matrix **S** is a design matrix that linearly transforms from the energy grid of the prior to the grid of the experiment.

Because energy-dependent discrepancies are observed between experiments, we build a Bayesian model to attempt to learn these discrepancy terms from the observed data, incorporating experimental metadata. For PFNS, there are many different metadata feature categories that may be related to observed discrepancies, each of which has several possible levels. For example, the metadata feature category “Neutron Detector Type” may have levels “Li-6”, “^235^U (n,f) chamber”, “SCIN (liquid)” or “Unknown”. To capture the relationship between metadata and systematic bias, we incorporate energy-dependent bias terms for each level of the feature. A group of data with a common level *c* is thus described as:

2 $${{{{\mathrm{\chi }}}}}_{c}^{E}={{{S}}}_{{{{\bf{c}}}}}{{{{\mathrm{\chi }}}}}^{T}\cdot {e}^{{\delta }_{c}}+{{{\mathbf{\varepsilon }}}},$$

3 $${\delta }_{c}={\gamma }_{c}{{{\bf{B}}}},$$

where the systematic discrepancy ${e}^{{\delta }_{c}}$ is modeled with the bias basis matrix **B** and the bias coefficient *γ*_*c*_. This multiplicative bias allows the observed mean for experimental observations with level *c* to be shifted upward or downward from the true PFNS curve, *χ*^*T*^. The bias coefficients, *γ*_*c*_, control the strength of the observed bias along the y-axis. Columns of **B** represent basis functions for different locations and length scales of potential bias along the x-axis as described in Eq. (4) of ref. ^6^. For instance, in Fig. 3 short, medium and long range basis functions are shown that define at what energies the bias is seen in a specific data set. Together **B** and *γ*_*c*_ identify the location and strength of deviations of specific data from the bulk of other experiments.

To estimate these metadata-informed biases ${e}^{{\delta }_{c}}$, we perform Bayesian inference to obtain posterior distributions for *γ*_*c*_ and *χ*^*T*^. This posterior is obtained by combining a sparsity-inducing prior distribution with experimental data and covariances using a sampling approach. This analysis is performed iteratively for one of the 41 metadata feature categories at a time using every time all 21 data sets. The resulting posterior distribution for one iteration identifies discrepancies in experimental data related to different levels (e.g., Li-6, ^235^U(n,f) chamber) of the specific feature category under investigation (e.g., Neutron Detector Type).

We use a horseshoe distribution as a prior for *γ*_*c*_ which is used in sparse regression problems where one has many potential explanatory predictors of which only a small fraction is anticipated to be useful. Using a horseshoe prior to encourage sparseness implements the parsimony principle of Ockham’s Razor^25^. It enforces a sparse number of significant biases and related features to be found and drives all other biases to *δ* ≈ 0. One advantage of Horseshoe regression is that near-redundant predictors can produce correlated posterior coefficients that retain the ambiguity of whether one or the other is the truly active variable. In essence, this prior avoids that statistical fluctuations in the data be misinterpreted as systematic discrepancy, and highlights few (sometimes correlated) metadata features as a potential source of bias.

The horseshoe prior for the bias coefficients *γ*_*c*_ is a hierarchical distribution that encourages sparsity. That means *γ*_*c*_ is described via a multi-level probability distribution, where its parameters themselves follow a probability distribution. Specifically, each coefficient *γ*_*c*_ follows:

4 $${\gamma }_{c} \sim {{{\mathcal{N}}}}(0,{\lambda }_{c}^{2}{\tau }^{2}),$$

5 $${\lambda }_{c} \sim {{\mbox{Cauchy}}}^{+}(0,1),\,\tau \sim {{{{\mathcal{N}}}}}^{+}(0,{\tau }_{0}^{2}),$$

where *λ*_*c*_ is a local shrinkage parameter for each coefficient and *τ* is the global shrinkage parameter that controls overall sparsity by setting a lower limit on how strong the evidence for a bias must be to not be shrunken to zero. The parameters *λ*_*c*_ and *τ* follow half-Cauchy and half-normal distributions, respectively, constrained to positive values. The Cauchy distribution for *λ*_*c*_ is sharply centrally peaked with tails that drop off slowly for large values. Due to this, this prior has substantial probability near zero, i.e., constraining *γ*_*c*_ near zero, while also allowing for a few large values corresponding to weakly informative prior information on *γ*_*c*_^5^. The parameter *τ*_0_ was chosen for this work such that discrepancies were identified as bias if more than 1% of the posterior samples of the bias term ${e}^{{\delta }_{c}}$ differed from unity (no bias) by more than 1%^6^.

The resulting posterior distribution for the bias coefficients *γ*_*c*_ and underlying nuclear data estimates χ^*T*^ are sampled using the No-U-Turn sampler in the Stan probabilistic programming language^26^. Some of these sample values are shown in the bottom panels of Fig. 3. We reiterate that this multi-scale fit is performed for each metadata feature and provides as output posterior distributions for *γ*_*c*_ and *χ*^*T*^.

After the Bayesian analysis, step three in the process in Fig. 1 is the subject matter expert review of the resulting posterior distribution for each *δ*_*c*_. Statistics of the posterior distribution, such as posterior probability of expression of bias in some energy region and visualization of the estimated bias functions, are shared with experts for analysis to determine plausible physical explanations for observed biases. Because there are many types of metadata categories and many experiments, several experiments may share levels of metadata features. For instance, a set of experiments that share the same level for neutron detector may also share the same level for a background correction or fission detector. This can lead to correlations between features that render bias identification purely from the data infeasible. In addition to that, the Bayesian model does not capture interactions between these experimental choices, because each metadata feature category is investigated independently. This is justified for PFNS experiments where many corrections can be approximated as multiplicative or linear functions as described in Section "PFNS Experiment Analysis". However, it also leads to missed correlations between feature categories and several feature categories being implicated in the same bias. This makes human-in-the-loop analysis essential for interpreting which explanations of bias is likely correct or requires deeper exploration via targeted future experiments or simulations.

### Regression analysis

Regression analysis with Generalized Least Squares is performed on χ^*E*^ and **Cov**^*E*^, to yield one recommended curve, χ^*R*^, that approximates truth χ^*T*^ with covariances, **Cov**^*R*^, based on the experimental database:

6 $${{{{\mathrm{\chi }}}}}^{R}={{{{\mathrm{\chi }}}}}^{P}+{{{{\bf{Cov}}}}}^{R}{({{{\bf{S}}}})}^{t}{({{{{\bf{Cov}}}}}^{E})}^{-1}\left({{{{\mathrm{\chi }}}}}^{E}-{{{\bf{S}}}}{{{{\mathrm{\chi }}}}}^{P}\right),\\ {{{{\bf{Cov}}}}}^{R}={{{{\bf{Cov}}}}}^{P}-{{{{\bf{Cov}}}}}^{P}{({{{\bf{S}}}})}^{t}{{{{\bf{Q}}}}}^{-1}{{{\bf{S}}}}{{{{\bf{Cov}}}}}^{P},$$

with

7 $${{{\bf{Q}}}}={{{\bf{S}}}}{{{{\bf{Cov}}}}}^{P}{({{{\bf{S}}}})}^{t}+{{{{\bf{Cov}}}}}^{E}.$$

For this procedure, we assume implicitly that *δ* = 0 in Eq. (1). The prior means, *χ*^*P*^, are the previous Standard mean values^24^ paired with prior covariances, **Cov**^*P*^, that are diagonal and have 100% uncertainty^7^. The matrix (**S**)^*t*^ is the transpose of the design matrix **S**. This algorithm is used to obtain “evaluated” data in Figs. 4 and 5 to assess the impact of clues found on the experimental databases in Fig. 1 by the Bayesian model on our understanding of the observable at hand.

### PFNS experiment analysis

**Fig. 6:**
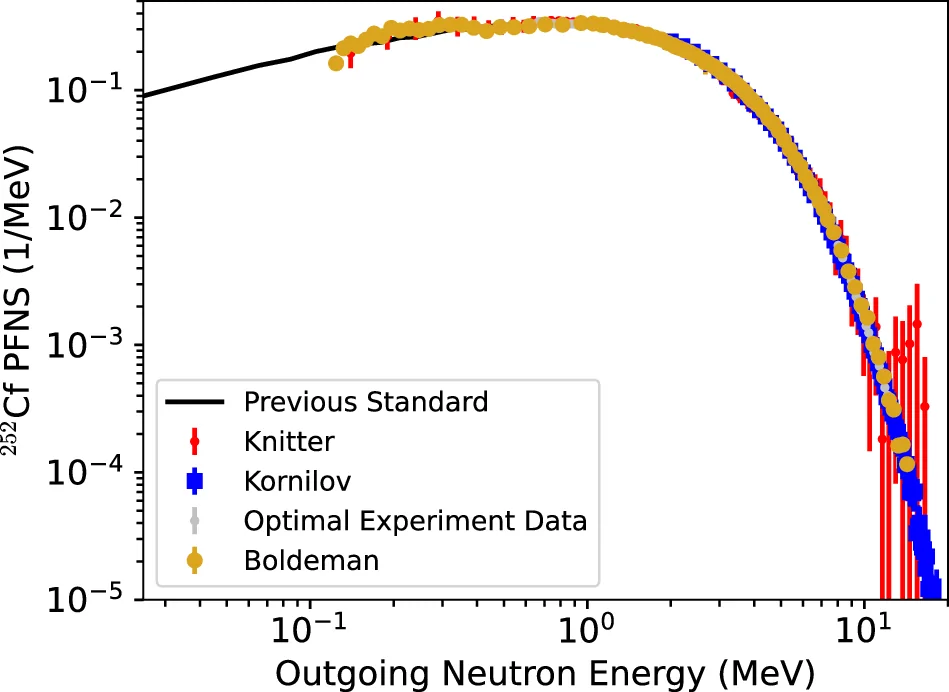
^252^Cf prompt fission neutron spectra in absolute scale. Selected ^252^Cf PFNS experiments are compared to the previous Standard (black solid line) in log-log scale to highlight the many orders of magnitude covered by this observable. Only those datasets are called out by name in the legend that are relevant for this work.

PFNS are reported as a function of outgoing neutron energy. These energies are obtained by using the relativistic time-to-energy transformation, $E={m}_{n}{c}^{2}({(1-{(\frac{L}{ct})}^{2})}^{-1/2}-1)$, where *t* and *L* are the time and length between fission and associated neutron detection, respectively, with a neutron mass *m*_*n*_ and the speed of light *c*. The PFNS, *χ*, is the result of complex analyses of measured counts *C* that are corrected for background counts *B* and the detector response function *R*, that vary with outgoing neutron energy *E*^12–14^:

8 $$\left[C({E}_{i})-B({E}_{i})\right]\kappa ({E}_{i})={\sum}_{j}R({E}_{i},{E}_{j})\times \chi ({E}_{j}),$$

where *E*_*i*_ is the measured energy, distorted by scattering effects, and *E*_*j*_ is the true energy of the emitted neutron. The variable *κ* captures any other correction (e.g., impurity in sample or deadtime) not included in *R*(*E*_*i*_, *E*_*j*_). The ^252^Cf PFNS has a maximum around approximately 2 MeV and falls then off by orders of magnitude as shown in Fig. 6. Due to that, all other spectra are shown here in ratio to a normalized Maxwellian function with temperature *T* = 1.42 MeV to highlight differences between data sets. Because the PFNS falls off rapidly in the wings, it is difficult to detect neutrons below 0.1 and above 8 MeV, where count-rates are extremely low and thus difficult to cleanly separate from considerable background counts. The other major challenge of PFNS measurements is to quantify accurate *R*(*E*_*i*_, *E*_*j*_) values as any mistake in that will directly translate into experimental bias. For our optimal experiment, *R* is determined by measuring with the same detector in the same environment other well-known observables, the ^12^C and ^9^Be elastic scattering reactions. Then, *R* is inferred by comparing counts *Y* from this measurement to the known value, *σ*, of the scattering reaction:

9 $$R({E}_{i})=Y({E}_{i})\eta ({E}_{i})/\sigma ({E}_{i}),$$

where *η* are further corrections of *Y* for backgrounds and other effects. For instance, neutrons might be converted in some detectors to *γ*s and one might not exactly know the light outputs for these conversions. Unfortunately, the higher *E* is the poorer the knowledge is on *σ* and *η* causing significant challenges above 10 MeV. In Eq. (9), the distinction between *E*_*i*_ and *E*_*j*_ is dropped by assuming that the measured and true energy are the same, an approximation often made in measurements. This approximation is valid for the optimal experiment because the component of *R*_*ij*_ from neutrons scattered in the environment has been removed from the analysis.

## Supplementary information

Description of Additional Supplementary File

Supplementary Data 1

Transparent Peer Review file

## Data Availability

All data generated or analyzed in this study are included as supplementary information files (named Supplementary Data 1).

## Code availability

The Bayesian model code is openly available as described in ref. ^27^. The code for the regression analysis^28^ is openly available as described in ref. ^29^.
