# Supplementary material for: Resolving root causes of experiment discrepancies guided by machine learning
Source: Nat Commun. 2026 Aug 27;17:10264. doi: 10.1038/s41467-026-76798-5 (PMC13616969; doi:10.1038/s41467-026-76798-5)
Supplement: Supplementary file 1 [file 41467_2026_76798_MOESM1_ESM.pdf]

Description of Additional Supplementary File

File Name – Supplementary Data 1

Description - This document describes the database related to a journal publication with title: "Resolving Root Causes of Experiment Discrepancies Guided by Machine Learning" [1]. This database is released as supplemental material to guarantee reproducibility. The supplemental material contains one folder with 21 files and 6 additional files:

- Four text files contain the output of nuclear data evaluations (outgoing energy, mean values and uncertainties) described in Ref. [1]. These are evaluated 252Cf prompt fission neutron spectra (PFNS) that are obtained via a regression analysis from experimental data described below.
- The folder contains 21 json files with experimental data described in Refs. [1, 2, 3] that were used as input for the machine learning analysis described in Ref. [1, 3]. These constitute experimental data by other authors over the last decades that have been published all in the open literature and were partially extracted through the openly accessibly EXFOR database [2] data. These data have been enhanced with experimental covariances using uncertainties from the open literature of the data and Ref. [4].
- The other two json databases are subsets of data described in Refs. [1, 2, 3, 5]. These also constitute experimental data over the last decades that have been published all in the open literature and were partially extracted through the openly accessibly EXFOR database [2] data.

Format:

- The evaluated files are simple text files with the first column containing outgoing energy (MeV), the second column the PFNS, and the third column relative uncertainties in %. The spectra are not normalized, and thus the PFNS has arbitrary units. These data can be read in python with a simple "loadtxt" statement, or in any text editor.
- The json experimental database contains the input data as used for the evaluations. The data can be read in python with a simple "json.load(jsonfile)" statement, or opened with a browser or text editor. Each dataset is contained in one dictionary that contains outgoing energy, mean values, covariances along with metadata key words extracted from the literature, EXFOR and documentation of thoughts of the authors.

References:

[1] Denise Neudecker, Keegan John Kelly, Scott Alan Vander Wiel, A.D. Carlson, Michael John Grosskopf, D.A. Brown, B. Pritychenko, Noah Anthony Wy Walton, and Robert Cameron

Haight. Resolving the root cause of experiment discrepancies guided by machine learning. Technical Report LA-UR-25-30840, Los Alamos National Laboratory, 2025.

[2] Denise Neudecker, Keegan John Kelly, Allan D. Carlson, Boris Pritychenko, David A. Brown, Michael John Grosskopf, Scott Alan Vander Wiel, Robert Cameron Haight, R. Capote, T. Massey, A. Trkov, and Noah Anthony Wy Walton. Re-evaluating the Prompt Fission Neutron Spectrum of Spontaneously Fissioning 252Cf. (LA-UR-24-24178), 2025. Accepted by EPJ-N.

[3] Noah A.W. Walton, Denise Neudecker, Scott A. Vander Wiel, Michael J. Grosskopf, and Keegan J. Kelly. Machine learning-assisted identification of potential sources of bias in measurements of prompt-fission neutron spectra. Computer Physics Communications, 315:109698, 2025.

[4] D. Neudecker, M. Devlin, R.C. Haight, K.J. Kelly, P. Marini, A.D. Carlson, J. Taieb, and M.C. White. Templates of expected measurement uncertainties for prompt fission neutron spectra. EPJ Nuclear Sciences & Technologies, 9:32, 2023.

[5] Keegan John Kelly, Denise Neudecker, Allan D. Carlson, Robert Cameron Haight, Michael John Grosskopf, Scott Alan Vander Wiel, Boris Pritychenko, Noah Anthony Wy Walton, Patrick Alexander Copp, Nicholas Andrew Mendez, and Jason Tyler Surbrook. Measurement of the 252Cf(sf) prompt fission neutron spectrum utilizing 12C(n,n) and 9Be(n,n) neutron scattering reference measurements. Technical Report LA-UR-25-31250, Los Alamos National Laboratory, 2025.
