# Supplementary material for: Resolving root causes of experiment discrepancies guided by machine learning
Source: Nat Commun. 2026 Aug 27;17:10264. doi: 10.1038/s41467-026-76798-5 (PMC13616969; doi:10.1038/s41467-026-76798-5)
Supplement: Supplementary file 2 [file 41467_2026_76798_MOESM3_ESM.pdf]

Peer Review File

Resolving Root Causes of Experiment Discrepancies Guided by Machine Learning

Corresponding Author: Dr Denise Neudecker

This file contains all reviewer reports in order by version, followed by all author rebuttals in order by version.

Version 0:

Reviewer comments:

Reviewer #1

(Remarks to the Author)
a) Summary and noteworthy results

This manuscript proposes a Bayesian, metadata-driven workflow to diagnose and resolve systematic discrepancies across multiple experiments measuring the same functional observable. The approach combines a hierarchical Bayesian functional model with sparse bias attribution to experimental metadata, and is demonstrated on the prompt fission neutron spectrum (PFNS) of (^{252})Cf, a long-standing benchmark with well-documented inter-experimental disagreement.

The most noteworthy results are the the identification of smooth, experiment-specific multiplicative bias functions, the attribution of dominant discrepancies to particular classes of experimental choices (notably detector response treatment), and the use of these findings to motivate data correction, selective rejection, and the design of a new experiment that is argued to be less correlated with historical biases.

b) Significance and originality

Within the field of nuclear data evaluation, the work is novel and potentially impactful. The integration of modern Bayesian sparsity techniques with experimental metadata to guide scientific investigation goes beyond standard evaluation pipelines and offers a principled alternative to ad hoc data selection or expert-only judgment.

However, the manuscript repeatedly frames the approach as a general method for resolving experimental discrepancies across science. At present, the evidence supports this claim only at the level of principle, not demonstration. All validation is performed on a single, highly favorable domain: a well-studied physical observable with rich metadata, known bias mechanisms, and the possibility of follow-up experimentation. As such, the contribution should be understood primarily as a strong proof-of-concept within nuclear physics rather than a broadly validated scientific workflow.

c= Support for conclusions and claims

The conclusions drawn about PFNS data quality and dominant sources of bias are plausible and consistent with prior knowledge in the field. The agreement between model-identified issues and known experimental limitations (e.g., detector response modeling) provides face validity.

That said, several conclusions—particularly those presented in Section 3.2—are supported mainly through qualitative narrative rather than quantitative Bayesian posterior summaries. The manuscript would benefit from clearer specification of how metadata–bias associations are assessed statistically (e.g., posterior inclusion probabilities, credible intervals, thresholds for importance), and whether alternative or null cases exist. Without this, it is difficult to assess how robust or general these conclusions are, as opposed to being selectively illustrative.

d) Data analysis, interpretation, and potential flaws

No obvious errors are apparent in the statistical formulation itself, which is technically sound and well motivated. However, there are serious limitations:

- Section 3.2 discusses only examples that align with known physics. It is unclear how representative these cases are, or whether contradictory or inconclusive cases exist.
- The direction of inference—whether the model independently identifies patterns later explained by metadata, or whether metadata guide interpretation post hoc—is not clearly articulated.

These issues do not prohibit publication, but they do warrant revision and clarification.

d) Methodology and modeling assumptions

The Bayesian methodology is coherent and appropriate for the stated goals. Importantly, the latent functon(f(E)) is modeled nonparametrically and does not assume linearity in energy. However, the bias attribution mechanism relies on a linear, additive decomposition of metadata effects in coefficient space. This implicitly assumes that systematic effects combine smoothly and additively, and does not capture nonlinear interactions between experimental choices.

This assumption is reasonable as a first-order approximation, but its implications for generality and robustness are not discussed. A brief discussion of this modeling choice, its limitations, and possible extensions would strengthen the paper.

e) Reproducibility and methodological detail

The largest weakness of the manuscript concerns insufficient methodological details, particularly regarding metadata construction and usage. Metadata are central to the model's explanatory power, yet key information is missing (e.g., how categorical feature levels are encoded in the design matrix? how missing or uncertain metadata are handled?)

Without fully detailed information, the analysis is difficult to reproduce or independently assess. At minimum, the paper would benefit from a supplementary table detailing all metadata features, levels, and encoding choices, or from making the metadata file publicly available.

f) Overall assessment

This is a potentially strong contribution that introduces a promising Bayesian framework for diagnosing experimental discrepancies, and it represents a meaningful advance for nuclear data evaluation. However, the manuscript currently overstates the generality of its conclusions and under-specifies key methodological components, particularly those related to metadata and the interpretation of model output.

I recommend major revision prior to publication, with emphasis on:

1. tempering or better qualifying claims of general applicability;
2. adding quantitative support and transparency to Section 3.2;
3. substantially expanding methodological detail on metadata construction.

(Remarks on code availability)

Reviewer #2

(Remarks to the Author)
What are the noteworthy results?

In this paper Neudecker and collaborators conduct a Bayesian analysis of the bias in a historical data set that includes the results of a number of different experiments which measured the spectrum of neutrons emitted in the prompt fission of 252Cf. These methods identify systematic bias in three data sets. In two out of three cases the bias an be associated with specific aspects of the neutron detection method employed. This is achieved because Neudecker et al. have carefully recorded metadata for each experiment; this enables a "rescue" of one of those sets of data. These efforts lead to a significant reduction in uncertainty of a fission standard, and revision of its central value, due to identification of flaws with a data set that would otherwise contribute significantly to the evaluation.

Will the work be of significance to the field and related fields? How does it compare to the established literature? If the work is not original, please provide relevant references.

The update of the prompt-fission neutron spectrum (PFNS) standard that Neudecker et al. are able to execute is useful and

important, but may not be of interest to scientists outside of nuclear physics and engineering. Section 3.4 does a nice job of explaining the impact that each aspect of the analysis carried out here has on the evaluation.

In my opinion the major scientific impact of this work is that it is a general methodology for analyzing discrepant data sets and identifying bias in one or more of them. Neudecker et al. demonstrate the efficacy of a Bayesian approach to the identification of bias in particular data sets. Their approach records metadata for each experiment and constructs a Bayesian model that fits the bias and associates it with features of the experiment. This method can be applied to a wide range of problems in nuclear physics where data sets need to be combined. And—as the authors point out—a method to combine data sets while removing or repairing biased ones has applications well beyond nuclear physics too.

I am not aware of any papers by other groups that have attempted identification of bias and the mechanisms that produced it by cross-data-set comparison and meta-data coding.

Does the work support the conclusions and claims, or is additional evidence needed?

Neudecker et al. have taken pains to explain their work in terms that will be accessible to the readers of Nature Communications. I am sure they are also constrained in the level of detail they can provide by the length limit of the journal.

However, this results is a paper in which many details are missing. For this reader, at least, more information is needed in order for me to understand, and trust, the analysis carried out here.

Specific details I would like added are:
- A list of the meta-data tags employed and a table of (or link to information on) which experiments were tagged with which features.

- Is the Sparse bias formalism applied to all experiments at once? This seems to be what the third panel of Fig. 1 implies. But, if that's the case, then I don't understand how identification of bias via this fit is tied to specific aspects of the metadata. Is this done a posteriori after the presence of short-scale, mid-scale, or long-scale bias is identified? Or is a separate sparse bias fit done for each meta-data tag, as Fig. 2 implies?

- I see there is a separate paper submitted (Ref. [5]) that gives the results of the optimal experiment that was identified as one that would grow confidence in this evaluation. In what sense was this experiment "optimal"? Also, the paragraph describing that experiment may be confusing to a non-specialist. What experiment was performed and how does it produce the points in grey in the upper-right panel of Fig. 1? Furthermore, a couple of sentences explaining how 9Be and 12C are used to define the detector response and why the information from the resulting measurement is then uncorrelated with previous efforts that used other reactions for that purpose would be helpful. Eventually I will (presumably) be able to go to Ref. [5] for answers to these questions, but some of the information should be presented here so that this paper can be an understandable and self-contained whole.

- More generally, a brief primer on neutron detection and its challenges would help non-nuclear-physicist readers understand why this ends up being the cause of so much grief in these attempts to reconcile different data sets. This would also help clarify certain stray neutron-detection details (e.g. "biased data (for the light output)" on p.7) that have crept in and are potentially distracting and confusing without more context.

I think it is particularly important to clarify these points, so that it is accessible to other researchers who are contemplating adapting this methodology to their specific context.

Are there any flaws in the data analysis, interpretation and conclusions? Do these prohibit publication or require revision?

Not as far as I can tell, but—as I said in answer to the previous question—I would like some more details on the analysis. This will require revision.

Is the methodology sound? Does the work meet the expected standards in your field?

I judge the methodology to be completely sound. This work markedly exceeds the standards of our field, since systematic errors that cause inconsistencies between data bedevil nuclear-physics analyses that combine data, and do so in analyses across energy scales from a few MeV to hundreds of GeV.

Is there enough detail provided in the methods for the work to be reproduced?
Not completely, but if certain aspects were added to the "Methods" section that would help greatly. See my comments above.

I do appreciate that a github link to the publicly available sparse_bias code is provided. This code is useful. But, without the additional code (and particularly the data sets) that represent its specific application for AIACHNE one cannot know exactly what was done.

Also, the data from the new "optimal experiment" are not presented here or (yet) publicly accessible as far as I can tell. This makes it technically impossible to reproduce the results.

In addition to the changes implied by the above answers to the questions asked by "Nature Communications", I would like the following more minor points addressed in the revised version of the manuscript:

- The abstract uses quite intense language to describe the situation: “crippling discrepancies”, “myriad possibilities”, “staggering factor of 6”. I would gently suggest that the authors tone it down a bit and let their very impressive results speak for themselves.

- Figure 1 is fine for a one-slide overview of the work, but it ends up being quite crowded and difficult to read. Can I suggest stacking the “before” and “after” panels vertically so they can be made larger? This could be done if the bias plot was presented as a separate figure. Otherwise this panel is very difficult to read. I also don’t think the picture of the chosen experiment adds very much information to the manuscript.

- A pedantic point about what is an experiment and what is not: in paragraph 1 the phrase “experimental data” is used. But some of these fields (e.g. meteorology) do not take experimental data but rather observations.

- It would be good to state the definition of “prompt neutrons” when the PFNS is first introduced on page 5.

- I gather that neutron time of flight is used to obtain the PFNS spectrum? If so, giving the formula for that, if only in the “Methods” section at the end, would provide an anchor point for readers who know kinematics but not nuclear physics.

- If space permits, it would be good to show the original PFNS spectrum somewhere so that a reader can appreciate the challenge involved in inferring it reliably over this energy range.

- It might help to mention that there are 21 distinct data sets already on p. 5 when the “collection of experiments” is first mentioned.

- Rather than “approximate the true 252Cf PFNS spectrum” don’t Neudecker et al. mean “infer the 252Cf PFNS spectrum”?

- Since the data inconsistencies shown in Figure 2 all occur at higher neutron energies would it be worth zooming in on that region, and perhaps using a linear scale for the x-axis?

- What is a “horseshoe prior”: is there space to define it in this paper?

If changes to address these points are implemented in a revised version of the manuscript it would be very suitable for "Nature Communications".

(Remarks on code availability)
I reviewed the repo, but did not attempt installing and running the code.

Reviewer #3

(Remarks to the Author)
This manuscript presents a clear and well-constructed framework for resolving long-standing discrepancies between independent experimental datasets using Bayesian ML techniques. Metadata were collected for 41 feature categories across 21 distinct datasets. By linking sparse bias functions to interpretable experimental metadata and validating the resulting inferences through both simulations and a targeted new experiment, the authors convincingly demonstrate how the spread across datasets in the Cf-252 PFNS database can be reduced by up to a factor of 6.
The work is well executed and clearly written, and its conceptual approach is relevant beyond nuclear physics.
Two clarifications and an additional question would strengthen the manuscript and enhance its readability.
It would be helpful to clarify how sensitive the identified bias features are to metadata completeness. In particular, how robust are the Bayesian model’s conclusions if certain metadata categories are missing or only coarsely defined?
It is stated that some uncertainties were augmented based on physics knowledge of PFNS experiments. Can you further explain the augmentation?
Question: Do the authors envision practical limits to scaling this approach to much larger databases with hundreds of experiments?

(Remarks on code availability)

Reviewer #4

(Remarks to the Author)
Dear authors,
this manuscript is interesting.
Nevertheless, as a general comment, figures should be redone to allow a better understanding of figure and text comments.
Figure 1 contains too much information and is called several times in the document .Perhaps having 2 figures is better ?
In addition, in the text a small description of the Bayesian method could be interesting even tough a much more detailed explanations could still be given in appendix.
Chapter 1 tries to explain the method but refers to Cf example which is shown in chapter 3...It make the paper difficult to read

in my opinion.
At last, the chapter 2 is strangely placed. It seems that eventhough the method is quite general, you first applies it to the spectrum data and then think that this is a general issue in experimental date analysis... A solution may be to merge chapter 1 and 2 as a general introduction of the method + the data requirements and then look in detail to the Cf example ?

(Remarks on code availability)
line 36-39 : Figure 1 is not very clear. Perhaps, it could be better to focus on the high energy part where the discrepancies are important ? It may be helpful for the text explanations. or have 2 or 3 figures ?
line 41 inconsistencies instead of uncertainty ?
line 43 : Replace application of science, such that we cannot eg." by "application of science. For example, we cannot ..." ?
line 57-60 : give some examples of " as a function of another quantity" : calibration, normalization ?
line 63,85 : again calls to figure 1...refers to Cf example given afterwards ...
line 64-73 ... add some mathematical description of what is done ?

chapter 2 is small and even-thought the method is general, your only application in the paper is for CF spectra... It may be a better idea to put this chapter (its contents) in chapter 1 ?
lien 109 : cf is also particularly
line 118-120 : Give credit to detectors (reference to chinu ?)
line 154-156 It may be helpful to add a dedicated figure instead to refering again to figure 1 !
line 175 again figure 1... add a dedicated figure
line 207 again figure 1 which is now 4 pages before.... It is not helping to understand the authors statements.
line 217-224 ....Already said in previous paragraphs why again ?
Conclusions : strange not to make any reference to the example which is driving all the statements of this paper? Perhaps insists on the fact that the studied example highlight generic issues ?

Version 1:

Reviewer comments:

Reviewer #1

(Remarks to the Author)
I thank the authors for their detailed response and for the revisions made to the manuscript. The revised version adequately addresses my previous comments. The authors have clarified the dependence of the method on metadata completeness and consistent feature definitions, provided a clearer explanation of the uncertainty augmentation procedure, and discussed practical aspects of scaling the approach to larger databases.

These additions improve the transparency and readability of the manuscript. I have no further major concerns. However, the code provided in the public repo some serious issues. See my comments below.

(Remarks on code availability)
The repository contains the main Python and Stan implementation, an example notebook, dependency files, and some tests. However, the README provides only limited installation guidance and does not explain how to reproduce the paper’s figures and results. The paper-specific data and complete analysis workflow are not included.

I was unable to run the complete code successfully. The dependency files are machine-specific, the notebook requires manual CmdStan configuration, and the test suite contains errors and incomplete tests. The code may be useful to experienced users as a starting point, but the paper’s results are only partially reproducible and the repository is not yet a fully usable community resource.

Reviewer #2

(Remarks to the Author)
I have read the revised version of “Resolving root causes of experiment discrepancies guided by machine learning” by Neudecker et al., and also the rebuttal to myself & the other referees that the authors have provided. The authors have done a good job of dealing with the various critiques raised during the first round of refereeing. I have only a fairly small number of remaining questions. I do also have some suggestions for corrections to English/expression and I believe I have found some typos.

First, the questions:
1. Are the biases plotted in Fig. 3 the combined effect of all biases identified for the different metadata features that were associated with those experiments? If so, how many different biases were found in each case?
2. Perhaps relatedly, the discussion at the bottom of p.11 says “…discrepancies were identified as bias if more than 1% of the samples showed more than 1% difference from the mean”. Samples of what?
3 I am still rather in the dark as to what the form of the "horseshoe prior" for gamma_c is. Can the prior distribution be quoted in the manuscript?
These questions perhaps point out that, in spite of the additional efforts by the authors, their workflow for identifying bias in

experimental data is still not completely clear—at least to this reader.

Now the typos/wording suggestions:
page 3, line 52: “observables” should be “observable”
page 3, lines 53-54: I would suggest “A separate posterior distribution is obtained for each feature value in a category. These results thus inform us which metadata values in a specific category are more likely to be related to bias.”
Lines 66-68: Wouldn’t it make sense to mention the hypothesis closer to the start of the paragraph?
page 3, lines 72-75: Here I would suggest “Because we obtain posterior distributions for one feature category at a time and there may be interplay between different features (aspects of the experimental setup) that leads to several of them being correlated with one another, the same bias might be attributed to values of a few feature categories. Therefore, it is vital that subject matter experts further interpret these posterior [not poster :)] distributions.”
Line 76: “Therefore, in the third step of the process, …”

page 5, line 106: I think the second “from fission” is not needed
Line 107: I would move the discussion of fission before the definition of the PFNS.

page 7, line 169: why not be more specific and say “The Bayesian model highlighted three possibilities”?
line 172 could then again reiterate that it is three possibilities that are being analyzed
lines 180-181: I would suggest “It should be emphasized that the key that led to salvaging the data were the limited number of possibilities identified by the Bayesian model.”

page 12, line 289: there is a typo “categoryg” should be “category”
page 13, line 339: how about saying “and several feature categories being implicated in the same bias”

Most of this second category of suggestions can be accepted or rejected at the authors’ discretion, although obviously the typos should be fixed.

(Remarks on code availability)

Reviewer #3

(Remarks to the Author)
The authors have addressed all comments and suggestions and implemented the requested corrections. I therefore recommend the paper for publication.

(Remarks on code availability)

Open Access This Peer Review File is licensed under a Creative Commons Attribution 4.0 International License, which permits use, sharing, adaptation, distribution and reproduction in any medium or format, as long as you give appropriate credit to the original author(s) and the source, provide a link to the Creative Commons license, and indicate if changes were

made.
In cases where reviewers are anonymous, credit should be given to 'Anonymous Referee' and the source.
The images or other third party material in this Peer Review File are included in the article’s Creative Commons license, unless indicated otherwise in a credit line to the material. If material is not included in the article’s Creative Commons license and your intended use is not permitted by statutory regulation or exceeds the permitted use, you will need to obtain permission directly from the copyright holder.
To view a copy of this license, visit https://creativecommons.org/licenses/by/4.0/

Dear editor and Reviewers #1-4,

We would like to thank you very much for the substantial time you took to review this paper, which was likely for some reviewers outside of their field. We are very thankful to get this feedback as we want to make this a paper that is readable for scientists beyond our field.

Below we answer point-by-point all issues raised by the reviewers.

Editor: Thank you again for submitting your manuscript "Resolving Root Causes of Experiment Discrepancies Guided by Machine Learning" to Nature Communications. We have now received reports from 4 reviewers and, based on their comments, we have decided to invite a revision of your work. Your revision should address all the points raised by our reviewers (see their reports below). The reviewers highlight that the method is presented as a generalist approach, yet it is validated only on a nuclear physics dataset. While we understand that identifying suitable additional datasets may be challenging, the manuscript would benefit from a more balanced presentation that reflects this limitation, alongside a clearer discussion of the method's potential constraints and its possible applications.

We did indeed try to find additional examples outside of nuclear physics and found a few data sets that looked initially promising. When we discussed how to implement the data, we soon realized that we would not be able to analyze the findings, because we are not subject matter experts. In fact, we would not be able to judge if a feature highlighted by ML would be plausible or not, nor would we be able to follow up with simulations and experiments outside of our field. Hence, we decided to rather present this work of proof-of-concept as suggested by Reviewer #1 that could be applicable beyond our field and tried to be more specific on requirements on metadata features.

REVIEWER COMMENTS

Reviewer #1 (Remarks to the Author):

a) Summary and noteworthy results

This manuscript proposes a Bayesian, metadata-driven workflow to diagnose and resolve systematic discrepancies across multiple experiments measuring the same functional observable. The approach combines a hierarchical Bayesian functional model with sparse bias attribution to experimental metadata, and is demonstrated on the prompt fission neutron spectrum (PFNS) of (^{252})Cf, a long-standing benchmark with well-documented inter-experimental disagreement.

The most noteworthy results are the the identification of smooth, experiment-specific multiplicative bias functions, the attribution of dominant discrepancies to particular classes of experimental choices (notably detector response treatment), and the use of these findings to motivate data correction, selective rejection, and the design of a new experiment that is argued to be less correlated with historical biases.

b) Significance and originality

Within the field of nuclear data evaluation, the work is novel and potentially impactful. The integration of modern Bayesian sparsity techniques with experimental metadata to guide scientific investigation goes beyond standard evaluation pipelines and offers a principled alternative to ad hoc data selection or expert-only judgment.

- However, the manuscript repeatedly frames the approach as a general method for resolving experimental discrepancies across science. At present, the evidence supports this claim only at the level of principle, not demonstration. All validation is performed on a single, highly favorable domain: a well-studied physical observable with rich metadata, known bias mechanisms, and the possibility of follow-up experimentation. As such, the contribution should be understood primarily as a strong proof-of-concept within nuclear physics rather than a broadly validated scientific workflow.

- We used the language of reviewer #1 to state that this is a proof of concept in the introduction at the beginning of the fourth paragraph (new text marked in red): "In this work, we show, as a proof-of-concept within the domain of nuclear physics, how the AIACHNE (AI/ML Informed cAlifornium CHi Nuclear data Experiment) team resolved detrimental experimental discrepancies; To this end, we applied} machine learning (ML) methods to answer the question:[…]"
- We also changed "Given these general input requirements, this approach for unraveling data discrepancies can be easily applied across many scientific subject areas." To (new is red): "Given these general input requirements, the approach presented here could be tested for use in subject areas with similar data structures."
- We also toned the general applicability down in the abstract.
- In section 2, we changed: "The broad process of Sec. 2 is applicable scientific disciplines and can be tailored to problem-specific needs. The basic data requirements are:" to (new is red): Execution of this process requires the following basic data inputs:"
- In the conclusion, we added: "We demonstrate this process as a proof of concept […]"
- In the conclusion, we added more qualifiers (new text in red): "In a nutshell, the knowledge gained on ^{252}Cf spectra through the process described here leads to more targeted experiments, faster progress in science related to ^{252}Cf spectra, and more efficient use of funding."
- In the conclusion, we replaced: "This process can be applied to a broad range of subject areas and requires as input experimental data and their uncertainties as a function of a continuous observable as well as descriptive metadata features for the experiments." With (new text int red): "One could apply this process to subject areas that can generate the required input, namely, several experimental data sets and their uncertainties as a function of a continuous observable as well as descriptive metadata features describing the experiments."

c= Support for conclusions and claims

The conclusions drawn about PFNS data quality and dominant sources of bias are plausible and consistent with prior knowledge in the field. The agreement between model-identified issues and known experimental limitations (e.g., detector response modeling) provides face validity.

That said, several conclusions—particularly those presented in Section 3.2—are supported mainly through qualitative narrative rather than quantitative Bayesian posterior summaries. The manuscript would benefit from clearer specification of how metadata–bias associations are assessed statistically (e.g., posterior inclusion probabilities, credible intervals, thresholds for importance), and whether alternative or null cases exist. Without this, it is difficult to assess how robust or general these conclusions are, as opposed to being selectively illustrative.

The association between metadata and bias is assessed through statistics of the posterior distribution for the basis function coefficients and through analysis of the resulting learned, energy-dependent bias functions. Because the sparsity can induce a bi-modal posterior, assessment of the posterior probability assigned to coefficients relating to a particular metadata feature that are above a relevance threshold can be used to identify data-indicated bias. These posterior probabilities can be communicated to subject matter experts who can review the resulting energy-dependent biases and their connection to levels of the metadata features.

Posterior samples can also be used to explore indications of multiple explanations for a relationship between metadata and bias. The posterior samples for basis function coefficients can be negatively correlated for different levels of a metadata feature (e.g. different types of fission detector) when the data support a bias for one level or the other, but not both. Assessment of posterior probability of expression of those coefficients can quantify the relative evidence that one detector or another may be an explanation for observed discrepancy, while the probability of neither coefficient being expressed can quantify evidence that the data show a statistically meaningful discrepancy at all.

We added also more text in section 1 and 4.1 on the Bayesian model and the Horseshoe prior in general.

To answer what metrics we have for the expert interpretation, we added at the end of Section 4.1: "After the Bayesian analysis, step three in the process in Fig.~\ref{fig:Cf252PFNS} is the subject matter expert review of the resulting posterior distribution for each δ_c. Statistics of the posterior distribution, such as posterior probability of expression of bias in some energy region and visualization of the estimated bias functions, are shared with experts for analysis to determine plausible physical explanations for observed biases."

We added more description on the horseshoe prior in Section 4.1 to give explicitly the threshold of importance: "The horseshoe prior for the bias coefficients γ has a global shrinkage parameter τ. This parameter controls overall sparsity by setting a lower limit on how strong the evidence for a bias must be to not be shrunken to zero. For the present work, τ follows a

half-normal distribution, $\tau \sim N+(0,\tau_0^2)$. The parameter τ_0 was chosen for this work such that discrepancies were identified as bias if more than 1\% of the samples showed more than 1\% difference from the mean~\cite{Walton:2025}."

Null cases (no effect, no difference) were observed under two conditions so far:

(1) if most of our data have unknown metadata feature. Therefore, we stated in the manuscript in the metadata requirements: "Data sets where large parts of the feature values are unknown, especially if the most important values are all unknown, should be rejected."

(2) If we enlarged uncertainties in experimental data to cover experimental discrepancies. We had one instance (Pu-239(n,f) cross sections), where we fed data into the ML algorithm, where the experimental data were artificially rendered consistent by adding uncertainties to all outlying data without physics justification. Of course, the ML algorithm did not find a bias in the data, but that was purely, because we effectively removed all biases by adding uncertainties to cover them. The text was changed to emphasize this by (red is new): "Discrepant measurement values of one quantity of interest as a function of another along with their uncertainties and correlations. Uncertainties should encompass all known sources of imprecision and potential bias but not be artificially enlarged to cover discrepancies."

Alternative cases do exist, where one bias is attributed to several reasons. We have noted that in the manuscript for Knitter and Kornilov data and answer this part of the question in the answer for the question right below.

d) Data analysis, interpretation, and potential flaws

No obvious errors are apparent in the statistical formulation itself, which is technically sound and well motivated. However, there are serious limitations:

- Section 3.2 discusses only examples that align with known physics. It is unclear how representative these cases are, or whether contradictory or inconclusive cases exist.

Yes, contradictory cases exist, and that is why a human-in-the-loop and follow-up experiments are important. We mention that in the original manuscript for Knitter data ("These were attributed by the Bayesian model to issues in the neutron detector response, background correction, and neutrons losing energy from interactions in the environment before hitting the neutron detector."), where multiple reasons are given by ML for discrepancy. While all of them are possible, we clearly state that we cannot correct them anymore and reject the data. So, what is inconclusive here is how to correct the data.

Another inconclusive example is discussed in the original manuscript with Kornilov data. The part that was inconclusive was that ML pointed us to several reasons for bias: "The Bayesian model highlighted potential issues in the fission fragment detection efficiency (see left-hand side of Fig.~\ref{fig:Kornilov_Knitter} where ML points to the gas used in the fission detector), the background correction, or the neutron detector response." The human expert then had to analyze which one is the biasing effect to correct the data.

The final key issue was whether we trust other experiments assessments with the detector response relying on H-1/U-235. Just because data agree does not mean there is no issue. They could just be biased in the same direction (which the ML algorithm would not find) and we miss an unknown physics bias. That is why we did the optimal experiment. Essentially, the optimal experiment is going after a potential unknown physics bias that we cannot validate otherwise.

The only other remaining inconclusive case that remained for the database, which we did not cover in this paper is two data sets at the highest energies (Maerten and Chalupka). Both data sets were disagreeing at the highest outgoing neutron energies. ML pointed us to fission chamber effects which we indeed could substantiate (again issues with underlying physics data and issues found in determining the detector efficiency) through simulations. This led to rejection of part of Maerten and Chalupka data that we could not trust (described in Denise Neudecker's EPJN article and Noah Walton's CPC article), which was very similar to the Kornilov example, and we therefore decided not to present. I call it inconclusive as at the end, we had to reject data we found out to be biased. Unfortunately, we were left with very few trustworthy experimental data points and had to ask the community to follow up with a measurement.

As these nuances on what was inconclusive at what stage of the analysis seemed to have been lost in the original text, we made changes to the language to make this clearer.

Kornilov example (new text in red): "Relying on the ML findings alone would have led to inconclusive results in as far as one does not know which ones of these few (very different) potential issues could be the sources of bias. Here, it was key that human experts followed up with a step-by-step analysis of […]"

Knitter example (new text in red): "These many major issues could not be conclusively corrected because of lack of measurement information."

For the optimal experiment we added: "While measurements relying on \$^1\$H and \$^{235}\$U reactions agree, this does not necessarily mean that these measurements are correct. They could just be biased in the same direction resulting in false confidence in the data."

- The direction of inference—whether the model independently identifies patterns later explained by metadata, or whether metadata guide interpretation post hoc—is not clearly articulated.

Thank you for pointing out that this is unclear in the manuscript. It indeed was and a second reviewer noted it as well. We assess biases in data for one feature category at a time but using the entire database of experimental data and covariances

We add in the methods section (4.1): "To estimate these metadata-informed biases \$e^{\delta_c}\$, we perform Bayesian inference to obtain posterior distributions for \$\gamma_c\$ and \$\chi^T\$. This posterior is obtained by combining a sparsity-inducing prior distribution with experimental data and covariances using a sampling approach. This analysis is performed iteratively for one of the 41 metadata feature categories at a time using every time all 21 data sets. The resulting posterior distribution for one iteration identifies discrepancies in

experimental data related to different levels (e.g., Li-6, ^{235}U(n,f) chamber) of the specific feature category under investigation (e.g., Neutron Detector Type)."

Then, we note that human experts are presented with all the posterior distributions for further exploration:

"We reiterate that this multi-scale fit is performed for each metadata feature and provides as output posterior distributions for γ_c and χ^T.}

After the Bayesian analysis, step three in the process in Fig.~\ref{fig:Cf252PFNS} is the subject matter expert review of the resulting posterior distribution for each δ_c. …"

These issues do not prohibit publication, but they do warrant revision and clarification.

d) Methodology and modeling assumptions

- The Bayesian methodology is coherent and appropriate for the stated goals. Importantly, the latent functon(f(E)) is modeled nonparametrically and does not assume linearity in energy. However, the bias attribution mechanism relies on a linear, additive decomposition of metadata effects in coefficient space. This implicitly assumes that systematic effects combine smoothly and additively, and does not capture nonlinear interactions between experimental choices.

This assumption is reasonable as a first-order approximation, but its implications for generality and robustness are not discussed. A brief discussion of this modeling choice, its limitations, and possible extensions would strengthen the paper.

We do indeed treat each metadata feature as a potential independent reason for bias, because each metadata feature is investigated independently as a potential source of bias. This is justified for PFNS experiments where many corrections can be approximated as multiplicative or linear functions as described in the Section on PFNS experiments. Investigating metadata categories separately, can lead to inconclusiveness when the results are combined for all features. Then, several single features could be the source of the same bias, and the human expert select which one is the correct one. This feature is certainly one key reason, why a human-in-the-loop is essential for interpreting results. We added the following text in Section 4.1: "Because there are many types of metadata categories and many experiments, several experiments may share levels of metadata features. For instance, a set of experiments that share the same level for neutron detector may also share the same level for a background correction or fission detector. This can lead to correlations between features that render bias identification purely from the data infeasible. In addition to that, the Bayesian model does not capture interactions between these experimental choices, because each metadata feature category is investigated independently. This is justified for PFNS experiments where many corrections can be approximated as multiplicative or linear functions as described in Section~\ref{ssec:method_PFNSexp}. However, it also leads to correlations between feature categories being missed and several feature categories being

related to the same bias. This makes human-in-the-loop analysis essential for interpreting which explanations of bias is likely correct or requires deeper exploration via targeted future experiments or simulations."

e) Reproducibility and methodological detail

Joint answer to:

- The largest weakness of the manuscript concerns insufficient methodological details, particularly regarding metadata construction and usage. Metadata are central to the model's explanatory power, yet key information is missing (e.g., how categorical feature levels are encoded in the design matrix? how missing or uncertain metadata are handled?)

- Without fully detailed information, the analysis is difficult to reproduce or independently assess. At minimum, the paper would benefit from a supplementary table detailing all metadata features, levels, and encoding choices, or from making the metadata file publicly available.

We provided in this review step supplemental material of all experimental data and their associated metadata features. These data will be provided also for the final publication as open access. The encoding of features is shown in these files explicitly, including the categorical feature levels. We use json files with key and values. When a specific metadata feature was not known, we listed it as unknown as can be seen in the submitted supplemental material. It is also described in the Methods Section (4.1) that each feature is analyzed separately in the algorithm. If something is still unclear, please do let us know.

f) Overall assessment

This is a potentially strong contribution that introduces a promising Bayesian framework for diagnosing experimental discrepancies, and it represents a meaningful advance for nuclear data evaluation. However, the manuscript currently overstates the generality of its conclusions and under-specifies key methodological components, particularly those related to metadata and the interpretation of model output.

I recommend major revision prior to publication, with emphasis on:

1. tempering or better qualifying claims of general applicability;

We tempered the language down in the abstract, introduction, Section 1 and Conclusion as described above.

2. adding quantitative support and transparency to Section 3.2;

We added as supplemental material all input data and metadata. We also sharpened the language more where the ML results on their own are inconclusive and we failed to correct data.

3. substantially expanding methodological detail on metadata construction.
We added supplemental material of all metadata and for the presented study. If something else beyond that is required to address this concern, please do let us know.

Reviewer #2 (Remarks to the Author):

What are the noteworthy results?

In this paper Neudecker and collaborators conduct a Bayesian analysis of the bias in a historical data set that includes the results of a number of different experiments which measured the spectrum of neutrons emitted in the prompt fission of 252Cf. These methods identify systematic bias in three data sets. In two out of three cases the bias an be associated with specific aspects of the neutron detection method employed. This is achieved because Neudecker et al. have carefully recorded metadata for each experiment; this enables a “rescue” of one of those sets of data. These efforts lead to a significant reduction in uncertainty of a fission standard, and revision of its central value, due to identification of flaws with a data set that would otherwise contribute significantly to the evaluation.

Will the work be of significance to the field and related fields? How does it compare to the established literature? If the work is not original, please provide relevant references.

The update of the prompt-fission neutron spectrum (PFNS) standard that Neudecker et al. are able to execute is useful and important, but may not be of interest to scientists outside of nuclear physics and engineering. Section 3.4 does a nice job of explaining the impact that each aspect of the analysis carried out here has on the evaluation.

In my opinion the major scientific impact of this work is that it is a general methodology for analyzing discrepant data sets and identifying bias in one or more of them. Neudecker et al. demonstrate the efficacy of a Bayesian approach to the identification of bias in particular data sets. Their approach records metadata for each experiment and constructs a Bayesian model that fits the bias and associates it with features of the experiment. This method can be applied to a wide range of problems in nuclear physics where data sets need to be combined. And—as the authors point out—a method to combine data sets while removing or repairing biased ones has applications well beyond nuclear physics too.

I am not aware of any papers by other groups that have attempted identification of bias and the mechanisms that produced it by cross-data-set comparison and meta-data coding.

Does the work support the conclusions and claims, or is additional evidence needed?

Neudecker et al. have taken pains to explain their work in terms that will be accessible to the readers of Nature Communications. I am sure they are also constrained in the level of detail they can provide by the length limit of the journal.

However, this results is a paper in which many details are missing. For this reader, at least, more information is needed in order for me to understand, and trust, the analysis carried out here.

Specific details I would like added are:
- A list of the meta-data tags employed and a table of (or link to information on) which experiments were tagged with which features.

We provided in this review step supplemental material of all experimental data and their associated metadata features. These data will be provided also for the final publication.

- Is the Sparse bias formalism applied to all experiments at once? This seems to be what the third panel of Fig. 1 implies. But, if that's the case, then I don't understand how identification of bias via this fit is tied to specific aspects of the metadata. Is this done a posteriori after the presence of short-scale, mid-scale, or long-scale bias is identified? Or is a separate sparse bias fit done for each meta-data tag, as Fig. 2 implies

Thank you for pointing out that this is unclear in the manuscript. It indeed was and a second reviewer noted it as well.

Yes, we apply the bias algorithm to all experimental data and covariances at once but for one feature category at a time.

We add in the methods section (4.1): "To estimate these metadata-informed biases e^{δ_c}, we perform Bayesian inference to obtain posterior distributions for γ_c and χ^T. This posterior is obtained by combining a sparsity-inducing prior distribution with experimental data and covariances using a sampling approach. This analysis is performed iteratively for one of the 41 metadata feature categories at a time using every time all 21 data sets. The resulting posterior distribution for one iteration identifies discrepancies in experimental data related to different levels (e.g., Li-6, ^{235}U(n,f) chamber) of the specific feature category under investigation (e.g., Neutron Detector Type)."

Joint answer to:
- I see there is a separate paper submitted (Ref. [5]) that gives the results of the optimal experiment that was identified as one that would grow confidence in this evaluation. In what sense was this experiment "optimal"? Also, the paragraph describing that experiment may be confusing to a non-specialist. What experiment was performed and how does it produce the points in grey in the upper-right panel of Fig. 1? Furthermore, a couple of sentences explaining how 9Be and 12C are used to define the detector response and why the information from the resulting measurement is then uncorrelated with previous efforts that used other reactions for that purpose would be helpful. Eventually I will (presumably) be able to go to Ref. [5] for answers to these questions, but some of the information should be presented here so that this paper can be an understandable and self-contained whole.

- More generally, a brief primer on neutron detection and its challenges would help non-nuclear-physicist readers understand why this ends up being the cause of so much grief in these attempts to reconcile different data sets. This would also help clarify certain stray neutron-detection details (e.g. “biased data (for the light output)” on p.7) that have crept in and are potentially distracting and confusing without more context.

As the physics of this measurement seems to be confusing in its currently written form, we pulled part of the experiment description out of the main text and added more in-depth description in the Methods section as suggested by the reviewer. This way it does not distract from the main flow of the article. The section explains how the data points seen in the plots are produced in general and briefly touches on the term light output. The added section is also meant to clarify how the detector response is determined which should make it clearer how 9Be and 12C are used to define the detector response. If still unclear, please do let us know. Here is the new section:

“PFNS are reported as a function of outgoing neutron energy. These energies are obtained by using the relativistic time-to-energy transformation, $E = m_nc^2 ((1 - (\frac{L}{ct})^2)^{-1/2} - 1)$, where t and L are the time and length between fission and associated neutron detection, respectively, with a neutron mass m_n and the speed of light c. The PFNS, χ, is the result of complex analyses of measured counts C that are corrected for background counts B and the detector response function R, that vary with outgoing neutron energy E~\cite{neudecker2023PFNStemplates,Capote:2016,Kelly:2020}:

\left [C(E_i)& -B(E_i) \right]\kappa(E_i) = \sum_{j} R(E_i,E_j) & \times \chi(E_j) ,\\

where E_i is the measured energy, distorted by scattering effects, and E_j is the true energy of the emitted neutron. The variable κ captures any other correction (e.g., impurity in sample or deadtime) not included in $R(E_i,E_j)$. The ^{252}Cf PFNS has a maximum around approximately 2~MeV and falls then off by orders of magnitude as shown in Fig.~\ref{fig:PFNSexample}. Due to that, all other spectra are shown here in ratio to a normalized Maxwellian function with temperature $T=$1.42 MeV to highlight differences between data sets. Because the PFNS falls off rapidly in the wings, it is difficult to detect neutrons below 0.1 and above 8 MeV, where count-rates are extremely low and thus difficult to cleanly separate from considerable background counts. The other major challenge of PFNS measurements is to quantify accurate $R(E_i,E_j)$ values as any mistake in that will directly translate into experimental bias. For our optimal experiment, R is determined by measuring with the same detectors in the same environment other, well-known observables, the ^{12}C and ^{9}Be elastic scattering reactions. Then, R is inferred by comparing counts, Y, from this measurement to the known value, σ, of the scattering reaction:

R(E_i)=Y(E_i)\eta(E_i)/\sigma(E_i),

where η are further corrections of Y for backgrounds and other effects. For instance, neutrons might be converted in some detectors to γs and one might not exactly know the light outputs for these conversions. Unfortunately, the higher E is the poorer the knowledge is on σ and η causing significant challenges above 10 MeV. In Eq.~\eqref{eq:responsefunction} the distinction between E_i and E_j is dropped by

assuming that the measured and true energy are the same, an approximation often made in measurements.This approximation is valid for the optimal experiment because the component of R_{ij} from neutrons scattered in the environment have been removed from the analysis.

The following text was added (new text in red) to explain in what sense the experiment was optimal and what was measured. The added text also tries to explain more how it is uncorrelated:

Therefore, the goal of our optimal experiment was to validate whether experiments relying on ^{1}H and ^{235}U reactions to determine the detector response can be trusted as this was the major outstanding question that could have led to large biases in data. Our ^{252}Cf PFNS experiment was optimal in as far as it was designed~\cite{Kelly2025Cf252PFNS} to be as uncorrelated with past experiments as possible for this feature. Hence, neither ^{1}H, ^{6}Li nor ^{235}U reaction were used to determine the detector response. Instead, elastic scattering reactions on ^{9}Be and ^{12}C were utilized for the very first time for ^{252}Cf PFNS to define the detector response in an energy range where the bulk of experimental ^{252}Cf PFNS exists.

The paper is indeed accepted by Physical review C at this point. We are happy to provide it to the editor to pass it on to the reviewer if needed.

I think it is particularly important to clarify these points, so that it is accessible to other researchers who are contemplating adapting this methodology to their specific context.

Are there any flaws in the data analysis, interpretation and conclusions? Do these prohibit publication or require revision?

Not as far as I can tell, but—as I said in answer to the previous question—I would like some more details on the analysis. This will require revision.

Is the methodology sound? Does the work meet the expected standards in your field?

I judge the methodology to be completely sound. This work markedly exceeds the standards of our field, since systematic errors that cause inconsistencies between data bedevil nuclear-physics analyses that combine data, and do so in analyses across energy scales from a few MeV to hundreds of GeV.

Is there enough detail provided in the methods for the work to be reproduced?
Not completely, but if certain aspects were added to the “Methods” section that would help greatly. See my comments above.

- I do appreciate that a github link to the publicly available sparse_bias code is provided. This code is useful. But, without the additional code (and particularly the data sets) that represent its specific application for AIACHNE one cannot know exactly what was done.

We apologize; the data set was not yet released at the time of submission but is released now. We will submit along with the re-submission. The release of the generalized-least squares code is in progress.

- Also, the data from the new “optimal experiment” are not presented here or (yet) publicly accessible as far as I can tell. This makes it technically impossible to reproduce the results.

We apologize; the data set was not yet released at the time of submission but is released now. We will submit along with the re-submission.

- In addition to the changes implied by the above answers to the questions asked by "Nature Communications", I would like the following more minor points addressed in the revised version of the manuscript:

- The abstract uses quite intense language to describe the situation: “crippling discrepancies”, “myriad possibilities”, “staggering factor of 6”. I would gently suggest that the authors tone it down a bit and let their very impressive results speak for themselves.

Thank you for calling that out. The new abstract is below and was also shortened to be within the 150-word limit of the journal:

Scientists rely on accurate experimental data to explain nature and then harness this knowledge for applications addressing human needs. However, discrepancies between experiments of the same observable can impede scientific progress if one does not understand the underlying causes. Here, we developed a process that unravels data discrepancies by first using Bayesian machine learning to relate discrepancies to few, of many, potentially biasing metadata features that encode experiment procedures. This machine learning output guides human experts to study discrepancy causes by simulating suspicious aspects of historical experiments or designing modern ones to address open questions. The study findings then lead to rejecting or correcting historical data on firm scientific bases. This process is demonstrated for the energy spectrum of neutrons emitted promptly ($<$~1~ns) after fission of ^{252}Cf, a trusted nuclear physics ``Standard". It reduced the spread in experimental ^{252}Cf spectra by up to a factor of 6.

- Figure 1 is fine for a one-slide overview of the work, but it ends up being quite crowded and difficult to read. Can I suggest stacking the “before” and “after” panels vertically so they can be made larger? This could be done if the bias plot was presented as a separate figure. Otherwise this panel is very difficult to read. I also don’t think the picture of the chosen experiment adds very much information to the manuscript.

The picture of the experiment was removed and references in the text to this part of the figure were removed and instead cited to previous journal articles: “Example pictures of hardware used to perform these experiments can be found in \cite{Kelly2025Cf252PFNS,Kelly:2021,Kelly_2022_235U}.”

Caption of Figure 1: “The process to resolve discrepancies in an experimental database: Starting from discrepant experimental data (top), ML methods provide clues as to what their root cause

could be. These clues then trigger simulations correcting historical data and designs of new experiments providing further validation. Thus, we attain our goal to resolve discrepancies in the database (bottom) that ultimately fuels scientific progress. Only those datasets are called out by name in the legend that are relevant for this work."

The bias identification plot is now a separate Figure (Fig. 2) and Fig. 1 has now the before and after panels vertically stacked.

Small changes have been made throughout the manuscript to correctly link to the changed figures.

- A pedantic point about what is an experiment and what is not: in paragraph 1 the phrase "experimental data" is used. But some of these fields (e.g. meteorology) do not take experimental data but rather observations.

We understand under experiment a designed set-up that is carefully controlled to isolate cause–effect relationships. On the other hand, an observation passively records what occurs naturally, like temperature and wind strength on any given day in a specific location.

All our 252Cf PFNS data are indeed experimental data as per the definition above. However, the methodology can also be applied to observations as the reviewer correctly points out. So, we added the term observation into the first paragraph.

I mark in red where observation was inserted:

Resulting experimental data or observations are key to understanding fundamental principles of nature, calibrating models of these natural processes, and then harnessing our resulting understanding to craft new applications that address human needs. Examples of subject areas that rely on accurate experimental data or observations …

- It would be good to state the definition of "prompt neutrons" when the PFNS is first introduced on page 5.

On page 5, the sentence was changed to:

The PFNS is the energy distribution of promptly emitted, that is within ($<$~1~ns) after fission, neutrons from fission …

- I gather that neutron time of flight is used to obtain the PFNS spectrum? If so, giving the formula for that, if only in the "Methods" section at the end, would provide an anchor point for readers who know kinematics but not nuclear physics.

As the physics of this measurement seems to be confusing in its currently written form, we pulled part of the experiment description out of the main text and added more in-depth description in the Methods section as suggested by the reviewer. This way it does not distract from the main flow of the article.

This sentence was added in 3.6.3:

"PFNS are reported as a function of outgoing neutron energy.

These energies are obtained by using the relativistic time-to-energy transformation, $E = m_nc^2 ((1 - (\frac{L}{ct})^2)^{-1/2} - 1)$, where t and L are the time and length between fission and associated neutron detection, respectively, with a neutron mass m_n and the speed of light c.

- If space permits, it would be good to show the original PFNS spectrum somewhere so that a reader can appreciate the challenge involved in inferring it reliably over this energy range.

Figure 6 was added with 252Cf PFNS in log-log scale to highlight the many orders of magnitude a PFNS covers.

- It might help to mention that there are 21 distinct data sets already on p. 5 when the "collection of experiments" is first mentioned.

The number of data sets was added as described on p. 5.

- Rather than "approximate the true 252Cf PFNS spectrum" don't Neudecker et al. mean "infer the 252Cf PFNS spectrum"?

The text was changed as suggested.

- Since the data inconsistencies shown in Figure 2 all occur at higher neutron energies would it be worth zooming in on that region, and perhaps using a linear scale for the x-axis? [I assume for Fig. 2, needs help from Noah]

Plots in Fig. 2 were changed to linear scale and zoomed in.

- What is a "horseshoe prior": is there space to define it in this paper?

We added the following paragraph in the Methods (Section 4.1) on how we implemented the horseshoe prior (the new text is highlighted in red):

We use a horseshoe distribution as a prior for γ_c which is used in sparse regression problems where one has many potential explanatory predictors of which only a small fraction is anticipated to be useful. Using a horseshoe prior to encourage sparseness implements the parsimony principle of Ockham's Razor~\cite{Ockham}. It enforces a sparse number of significant biases and related features to be found and drives all other biases to $\delta \approx 0$. One advantage of Horseshoe regression is that near-redundant predictors can produce correlated posterior coefficients that retain the ambiguity of whether one or the other is the truly active variable. In essence, this prior avoids that statistical fluctuations in the data be misinterpreted as systematic discrepancy, and highlights few (sometimes correlated) metadata features as a potential source of bias.

The horseshoe prior for the bias coefficients γ has a global shrinkage parameter τ. This parameter controls overall sparsity by setting a lower limit on how strong the evidence for a bias must be to not be shrunken to zero. For the present work, τ follows a half-normal distribution, $\tau \sim N+(0,\tau_0^2)$. The parameter τ_0 was chosen for this work such that discrepancies were identified as bias if more than 1\% of the samples showed more than 1\% difference from the mean~\cite{Walton:2025}.

If changes to address these points are implemented in a revised version of the manuscript it would be very suitable for "Nature Communications".

Reviewer #2 (Remarks on code availability):

I reviewed the repo, but did not attempt installing and running the code.

Reviewer #3 (Remarks to the Author):

This manuscript presents a clear and well-constructed framework for resolving long-standing discrepancies between independent experimental datasets using Bayesian ML techniques. Metadata were collected for 41 feature categories across 21 distinct datasets. By linking sparse bias functions to interpretable experimental metadata and validating the resulting inferences through both simulations and a targeted new experiment, the authors convincingly demonstrate how the spread across datasets in the Cf-252 PFNS database can be reduced by up to a factor of 6.
The work is well executed and clearly written, and its conceptual approach is relevant beyond nuclear physics.
Two clarifications and an additional question would strengthen the manuscript and enhance its readability.

- It would be helpful to clarify how sensitive the identified bias features are to metadata completeness. In particular, how robust are the Bayesian model's conclusions if certain metadata categories are missing or only coarsely defined?
All metadata categories should be the same across all experiments, even if they might be not applicable (say a specific correction might not be necessary for certain set-up but necessary for others; even in the former case, this feature value should be "N/A"). These categories should encompass all features that could lead to bias in an experiment. If for a specific experiment, the value for a feature category is not known, it should be stated as "unknown". We rejected data sets from the database where large amounts of values for necessary categories were unknown (e.g., data sets without any knowledge on corrections or set-up description), as one would not be able to draw any conclusion on the biasing factors in these experiments. However, as one can observe in the supplementary material, about one fifth to one fourth of the values for the feature categories of each individual experimental data set was unknown, and the algorithm still worked well. The reason for that is likely that we implicitly sorted out any data set that was missing

crucial information. However, the algorithm can also highlight as source of bias that is unknown if, for instance, a correction was undertaken. This information is still helpful for us as it could tell us to question the data given that we don't know if a correction was undertaken which could potentially lead to bias.

Too coarsely (or actually ambiguously) defining the feature categories can have detrimental impact. For instance, we found out that just adopting the detector descriptors from the EXFOR database was not informative enough, because they don't list the active material in the detector. To be more specific, Lajtai, Bao and Boldeman use Li-6 as active material for their neutron detectors, but they are categorized as GLASD versus SCIN (the coarse category of detector that does not speak to the detail of what material is used) in EXFOR which was not helpful in finding bias. It was a critical step to make the database AI/ML-ready in as far as the same quantity has exactly the same name across all experiments. For this example, we needed to call out that Li-6 is in the detector in addition to GLASD and SCIN. That is why it is important to define at the beginning what metadata categories could be impactful and how to describe them in the same terms across all experiments.

In section 1, we added for the data requirements:

"It is key that one defines at first all feature categories that could lead to biases in the studied data to enable the Bayesian model identifying biases. In addition, one must guarantee that metadata are easily interpretable by the Bayesian model which requires utilizing a nomenclature to catalog feature categories and names that is consistent across all data sets. If for a specific experiment, the value of a feature value is either unknown or not applicable, this should be stated as such. Data sets where large parts of the feature values are unknown, especially if the most important values are all unknown, should be rejected."

- It is stated that some uncertainties were augmented based on physics knowledge of PFNS experiments. Can you further explain the augmentation?

For instance, no uncertainties for impurities in the sample, that could result in a mixture of PNFS from several materials rather than just ^{252}Cf, were found for the experiment related to Ref.~\cite{Maerten:1990}.

Hence, covariances for this (small) effect were estimated based on a broad review of PFNS experiment uncertainty~\cite{neudecker2023PFNStemplates} and added in quadrature to the known covariances for this measurement.

- Question: Do the authors envision practical limits to scaling this approach to much larger databases with hundreds of experiments?
We are currently scaling this algorithm to include on the order of a few hundreds of experiments (roughly 200-300). While the Bayesian model takes longer to run, we have not encountered any major issue that would lead us to conclude that it would fail for larger databases. The number of parameters in the model (the basis function coefficients) are the larger scaling concern and an area of future work.

Reviewer #4 (Remarks to the Author):

Dear authors,
this manuscript is interesting.
Nevertheless, as a general comment, figures should be redone to allow a better understanding of figure and text comments.

- Figure 1 contains too much information and is called several times in the document .Perhaps having 2 figures is better ?

Thank you for the comment. Another reviewer also pointed that out. We simplified Fig. 1 by removing the experiment picture entirely and making the bias plot highlighting issues in Boldeman data a separate plot (now Fig. 2).

The picture of the experiment was removed and references in the text to this part of the figure were removed and instead cited to previous journal articles: "Example of pictures hardware used to perform these experiments can be found in \cite{Kelly2025Cf252PFNS,Kelly:2021,Kelly_2022_235U}."

Caption of Figure 1: "The process to resolve discrepancies in an experimental database: Starting from discrepant experimental data (top), ML methods provide clues as to what their root cause could be. These clues then trigger simulations correcting historical data and designs of new experiments providing further validation. Thus, we attain our goal to resolve discrepancies in the database (bottom) that ultimately fuels scientific progress. Only those datasets are called out by name in the legend that are relevant for this work."

The bias identification plot is now a separate Figure (Fig. 2) and Fig. 1 has now the before and after panels vertically stacked as suggested by the other reviewer.

Small changes have been made throughout the manuscript to correctly link to the changed figures.

- In addition, in the text a small description of the Bayesian method could be interesting even tough a much more detailed explanations could still be given in appendix.

At the beginning of Section 2, we expanded the text a bit (new text in red):

This second step of the process uses a Bayesian model to determine for each feature category a posterior distribution for the strength of a potential bias. This posterior distribution has different values for each feature value in a category and, thus informs us which metadata value is more likely than another in a specific category to be related to bias. These} metadata encode intricate details of experiment makeup and analysis. The hypothesis here is that biases in measured data must stem from something inherent to the experiment---for instance, poorly understood equipment or incorrect analysis steps. We chose a horseshoe prior to sample the strength of potential biases with the Bayesian model and link them to specific metadata feature values as it

enforces sparseness so that not every statistical fluctuation is identified as bias and a single root cause is preferred to multiple interacting ones~\cite{carvalho2009handling}. The fitted Bayesian model highlights a sparse set of features that are potentially responsible for biases~\cite{Walton:2025}. Because we obtain posterior distributions for each feature category at a time and several of the features might be correlated with another, the same bias might be attributed to be related to values of a few feature categories. Therefore, it is vital that subject matter experts further interpret these poster distributions.

- Chapter 1 tries to explain the method but refers to Cf example which is shown in chapter 3...It make the paper difficult to read in my opinion.

In the text, we do not refer to Cf before Section 3 on purpose. Instead, we rather use the pictures of the database before and after the ML analysis in Fig. 1 as an unspecified example of discrepancies in the database and refer to the differently colored figures. Some readers could be more visual people, who would benefit from seeing a picture of the discrepancies and how they can be resolved.

We do agree with the reviewer that Figure 1 was previously overloaded and likely had too much Cf-252 PFNS physics in it. By removing the bias-identification figure and the photograph of the experiment set up, we reduced that and let the reader focus on database before and after. When the figure is now mentioned in chapter 1 no knowledge of Cf-252 PFNS is yet needed. We hope that this is acceptable.

- At last, the chapter 2 is strangely placed. It seems that eventhough the method is quite general, you first applies it to the spectrum data and then think that this is a general issue in experimental date analysis... A solution may be to merge chapter 1 and 2 as a general introduction of the method + the data requirements and then look in detail to the Cf example ? [See if that is an option]
We incorporated chapter 2 into chapter 1 as recommended.

Reviewer #4 (Remarks on code availability):

- line 36-39 : Figure 1 is not very clear. Perhaps, it could be better to focus on the high energy part where the discrepancies are important ? It may be helpful for the text explanations. or have 2 or 3 figures ?

We changed Fig. 1 as described above by splitting it into two figures and removing We chose to not to change the x axis to linear scale as the bias at 200 keV is also important for the paper. But later in Fig. 3 we switched to a logarithmic scale to improve visibility for the high-energy bias.

- line 41 inconsistencies instead of uncertainty ?

This question refers to sentence: “This translates into uncertainty when scientists (a) calibrate models to these experimental data, or (b) estimate the true curve for the natural phenomenon of interest.”

The reviewer means likely that it leads to inconsistency (*c*ontradiction between values) on the input data for the procedures described in (a), (b), while it was meant that data discrepancy results in uncertainty (lack of precision on result). As this sentence seems to be confusing, we reformulated it to: “Such experimental-data inconsistencies result in uncertainties on model parameterizations or estimates of the true curve for a natural phenomenon that are based on these experiments as input.”

- line 43 : Replace application of science, such that we cannot eg." by "application of science. For example, we cannot ..." ?

Implemented as suggested.

- line 57-60 : give some examples of " as a function of another quantity" : calibration, normalization ?

The following sentence was added:

For instance, an observables could be measured as a function of time or temperature with metadata describing the measurement instrument and location.

- line 63,85 : again calls to figure 1...refers to Cf example given afterwards ...

In the text, we do not refer to Cf before Section 3 on purpose. Instead, we rather use the pictures of the database before and after the ML analysis as an unspecified example of discrepancies in the database and refer to the differently colored figures. Some readers could be more visual people, who would benefit from seeing a picture of the discrepancies and how they can be resolved.

We do agree with the reviewer that Figure 1 was previously overloaded and likely had too much Cf-252 PFNS physics in it. By removing the bias-identification figure and the photograph of the experiment set up, we reduced that. When the figure is now mentioned in chapter 1 no knowledge of Cf-252 PFNS is yet needed. We hope that this is acceptable.

- line 64-73 ... add some mathematical description of what is done ?

Section 4.1 was extended with more mathematical description of the horseshoe prior. We kept Section 2. on purpose on a high level without Eqs to be easier accessible to the reader and then giving the Eqs. in the methods section 4.1. We also expanded at the beginning of Section 2 the description of the Bayesian model as recommended by reviewer #4.

- chapter 2 is small and even-thought the method is general, your only application in the paper is for CF spectra... It may be a better idea to put this chapter (its contents) in chapter 1 ?

What was previously Chapter 2 (Data Requirements for the Process to be Applicable) was embedded into Chapter 1.

- lien 109 : cf is also particularly

Implemented as suggested.

- line 118-120 : Give credit to detectors (reference to chinu ?)

Citations to references [5,16,17] were added.

- line 154-156 It may be helpful to add a dedicated figure instead to refering again to figure 1 !

Thank you for that suggestion. We split Fig. 1 apart and what is now Fig. 2 corresponds to the text in lines 154-156 following the reviewer's suggestion.

- line 175 again figure 1... add a dedicated figure

Here, we refer to the database without discrepancy. We decided to keep the discrepant database and the one with resolved discrepancies in one plot to highlight the improvement through this process.

- line 207 again figure 1 which is now 4 pages before.... It is not helping to understand the authors statements.

We removed one reference to Fig. 1 and wrote instead "[…] trust in the remaining historical database, where discrepancies were resolved." In addition to that, the optimal experimental data were added to what is now Fig. 5 to have a Figure closer to the description of the optimal experiment showing the resulting experimental data.

line 217-224 ....Already said in previous paragraphs why again ?

We removed this part entirely.

- Conclusions : strange not to make any reference to the example which is driving all the statements of this paper? Perhaps insists on the fact that the studied example highlight generic issues ?

Thank you for pointing that out. We added the following sentences: "We demonstrate this process as a proof of concept using 21 ^{252}Cf prompt fission neutron spectrum data sets; in

this case, the machine learning algorithm provided the clues to reduce the spread in the database by up to a factor of six.”

Dear editor and Reviewers #1-3,

We would like to thank you very much for the substantial time you took to review this paper. Below we answer point-by-point all issues raised by the reviewers.

First, we ask you to revise your paper to address our editorial requests (in the attached Author Checklist) and any remaining comments from reviewers (included at the end of this email, if applicable).

We addressed all editorial requests and the remaining comments from the reviewers below

REVIEWERS' COMMENTS

Reviewer #1 (Remarks to the Author):

I thank the authors for their detailed response and for the revisions made to the manuscript. The revised version adequately addresses my previous comments. The authors have clarified the dependence of the method on metadata completeness and consistent feature definitions, provided a clearer explanation of the uncertainty augmentation procedure, and discussed practical aspects of scaling the approach to larger databases.

These additions improve the transparency and readability of the manuscript. I have no further major concerns. However, the code provided in the public repo some serious issues. See my comments below.

Thank you for re-reading the manuscript! We appreciate the time you took for that.

Reviewer #1 (Remarks on code availability):

The repository contains the main Python and Stan implementation, an example notebook, dependency files, and some tests. However, the README provides only limited installation guidance and does not explain how to reproduce the paper's figures and results. The paper-specific data and complete analysis workflow are not included.

I was unable to run the complete code successfully. The dependency files are machine-specific, the notebook requires manual CmdStan configuration, and the test suite contains errors and incomplete tests. The code may be useful to experienced users as a starting point, but the paper's results are only partially reproducible and the repository is not yet a fully usable community resource.

Thank you for pointing that out. We (to be specific Noah Walton) are currently working on addressing this issue by bringing in the data of the supplemental material as a test example into the sparse_bias code that it is easier to explicitly run the example shown in the paper. We acknowledge that a manual cmdstan build is required to run the example, but there is not

currently a way around this. The cmdstan utility is a standalone software program that performs the MCMC sampling. Installation will vary by architecture and OS, so we cannot integrate this any further than it already is into the sparse_bias code. However, detailed build/install instructions are given in the cmdstan documentation which is linked in our readme.

In addition, we just obtained LANL's open-source approval for the second code (ARIADNE, used for the regression analysis). We (to be specific Denise Neudecker) are working currently on putting the code on github (https://github.com/lanl/ARIADNE) and used the data of the supplemental material as a test example (sub-folder "Examples/Cf-252/PFNS_EvaluationImpactAssessment") to show how to use the code but also to guarantee reproducibility of Figs. 4 and 5.

Reviewer #2 (Remarks to the Author):

I have read the revised version of "Resolving root causes of experiment discrepancies guided by machine learning" by Neudecker et al., and also the rebuttal to myself & the other referees that the authors have provided. The authors have done a good job of dealing with the various critiques raised during the first round of refereeing. I have only a fairly small number of remaining questions. I do also have some suggestions for corrections to English/expression and I believe I have found some typos.

Thank you for re-reading the manuscript! We appreciate the time you took for that and answer below the remaining questions.

First, the questions:
1. Are the biases plotted in Fig. 3 the combined effect of all biases identified for the different metadata features that were associated with those experiments? If so, how many different biases were found in each case?

I am not quite sure if I correctly understand the question. I attempt an answer here: The bias curves, broken down into the different length scale components of the multi-scale model (the short, medium, and long range bases), plotted in the bottom of Fig. 3 for Kornilov and Knitter data show the multiplicative bias learned for that dataset from identifying potential active bias terms e^{δ_c} for the entire database. We then connect each identified bias to several metadata categories and values. A subset of those identified metadata values is called out in the title of the plot. Several curves, corresponding to curves samples from the posterior distribution for the bias, are plotted translucently to communicate both the size and uncertainty in the bias component. As we discuss in the text, this combination of metadata category and value is just one potential explanation for the bias, but limited observations and confounding mean there are several others that we must use nuclear data expertise to then differentiate. For Kornilov data, 3 strong reasons were given as called out in the text. For Knitter, multiple scattering, background, fission and neutron detector efficiency were given as reasons with high probability from 2-20 MeV (as mentioned in the text). Boldeman was a category of its own with very high bias from 100-600 keV that are largely associated with the neutron detector (neutron detector response method, type, multiple scattering related to neutron detectors), but has also been grouped together with data sets that also employ a Li-6 glass detector.

As we did not yet describe what other feature values appeared for Boldeman data, a sentence was added to this end in Section 2.4: "Several other potential feature values were called out as explanation for the bias, the majority of them was tied to the neutron detector (such as how its response was determined, or scattering effects distorting this response)."

To call out more clearly that all (x,y) data are used to identify a bias term in the database, the following was added in Section 2.3:
In fact, the bottom of Fig.~\ref{fig:Boldeman} shows that the Bayesian model identified a significant medium-range bias term for Boldeman data compared to the mean estimated from the entire database.

2. Perhaps relatedly, the discussion at the bottom of p.11 says "…discrepancies were identified as bias if more than 1% of the samples showed more than 1% difference from the mean". Samples of what?

Samples of the posterior distribution are drawn. This posterior distribution quantifies how likely it is that a bias term exp(delta_c) with (with basis B and strength gamma) is present to explain the data as related to truth according to Eq. (2). If 1% of the samples differ by more than 1% from the mean, then a bias is predicted to be present.

The sentence was edited to (change in red): "The parameter τ_0 was chosen for this work such that discrepancies were identified as bias if more than 1\% of the posterior samples of the bias term e^{δ_c} differed from unity (no bias) by more than 1\%~\cite{Walton:2025}."

3 I am still rather in the dark as to what the form of the "horseshoe prior" for gamma_c is. Can the prior distribution be quoted in the manuscript?
These questions perhaps point out that, in spite of the additional efforts by the authors, their workflow for identifying bias in experimental data is still not completely clear—at least to this reader.

Thank you for pointing that out. We added some text to more clearly describe the distribution of gamma_c. We hope that this now clarifies the Bayesian model in Section 4.1 (new text in red):

"The horseshoe prior for the bias coefficients γ_c is a hierarchical distribution that encourages sparsity. That means that γ_c is described via a multi-level probability distribution, where its parameters themselves follow a probability distribution. Specifically, each coefficient γ_c follows:

$$\gamma_c \sim \mathcal{N}(0, \lambda_c^2 \tau^2),$$
$$\lambda_c \sim \text{Cauchy}^+(0,1), \ \tau \sim \mathcal{N}^+(0, \tau_0^2),$$

where λ_c is a local shrinkage parameter for each coefficient and τ is the global shrinkage parameter that controls overall sparsity by setting a lower limit on how strong the

evidence for a bias must be to not be shrunken to zero. The parameters λ_c and τ follow half-Cauchy and half-normal distributions, respectively, constrained to positive values. The Cauchy distribution for λ_c is sharply centrally peaked with tails that drop off slowly for large values. Due to this, this prior has substantial probability near zero, i.e., constraining γ_c near zero, while also allowing for a few large values corresponding to weakly informative prior information on γ_c~\cite{carvalho2009handling}.

Now the typos/wording suggestions:
- page 3, line 52: “observables” should be “observable”

Corrected, thank you!

- page 3, lines 53-54: I would suggest “A separate posterior distribution is obtained for each feature value in a category. These results thus inform us which metadata values in a specific category are more likely to be related to bias.”

We think reviewer 2 means lines 63-65. The posterior distribution is given for each feature category and all feature values in that category. Like “Neutron Detector Type” is the category and “IOCH” and “SCIN” are values. So, the first proposed sentence is not quite right. But the reviewer correctly points out that the sentence is rather long and hard to read. So, we updated lines 63-65 to read: “This posterior distribution has different values for each feature value in a category. These results thus inform us which metadata values in a specific category are more likely to be related to bias.”

- Lines 66-68: Wouldn’t it make sense to mention the hypothesis closer to the start of the paragraph?

That is a good point. We moved the sentences below to the end of the first paragraph of this section (sentences 3 and 4 of the section):

“The hypothesis here is that biases in measured data must stem from something inherent to the experiment---for instance, poorly understood equipment or incorrect analysis steps. Therefore, metadata encode intricate details of experiment makeup and analysis.”

- page 3, lines 72-75: Here I would suggest “Because we obtain posterior distributions for one feature category at a time and there may be interplay between different features (aspects of the experimental setup) that leads to several of them being correlated with one another, the same bias might be attributed to values of a few feature categories. Therefore, it is vital that subject matter experts further interpret these posterior [not poster :)] distributions.”

This was implemented.

- Line 76: “Therefore, in the third step of the process, …”

This was implemented.

- page 5, line 106: I think the second “from fission” is not needed

Thank you, yes, the word-repetition is not ideal. The sentence reads now: “The PFNS is the energy distribution of promptly emitted, that is within ($<$~1~ns), neutrons from fission” (the second fission is more needed for accuracy then the one right after 1 ns).

- Line 107: I would move the discussion of fission before the definition of the PFNS.

This makes sense and was implemented.

Combined answer to:
- page 7, line 169: why not be more specific and say “The Bayesian model highlighted three possibilities”?

- line 172 could then again reiterate that it is three possibilities that are being analyzed

The text was re-written as (the changes are red): “The Bayesian model highlighted three possibilities for issues: (1) in the fission fragment detection efficiency (see left-hand side of Fig.~\ref{fig:Kornilov_Knitter} where ML points to the gas used in the fission detector), (2) the background correction, or (3) the neutron detector response. Relying on the ML findings alone would have led to inconclusive results in as far as one does not know which ones of these three (very different) potential issues could be the sources of bias.”

- lines 180-181: I would suggest “It should be emphasized that the key that led to salvaging the data were the limited number of possibilities identified by the Bayesian model.”

This was implemented.

- page 12, line 289: there is a typo “categoryg” should be “category”

Corrected, thank you!

- page 13, line 339: how about saying “and several feature categories being implicated in the same bias”

This was implemented.

Most of this second category of suggestions can be accepted or rejected at the authors' discretion, although obviously the typos should be fixed.

Reviewer #3 (Remarks to the Author):

The authors have addressed all comments and suggestions and implemented the requested corrections. I therefore recommend the paper for publication.

Thank you very much for your second review!
